# Comprehensive Geriatric Assessment and Quality of Life Aspects in Patients with Recurrent/Metastatic Head and Neck Squamous Cell Carcinoma (HNSCC)

**DOI:** 10.3390/jcm12175738

**Published:** 2023-09-03

**Authors:** Anna Winter, Stefan M. Schulz, Marc Schmitter, Urs Müller-Richter, Alexander Kübler, Sylvia Kasper, Stefan Hartmann

**Affiliations:** 1 Department of Prosthodontics, University Hospital Würzburg, Pleicherwall 2, 97070 Würzburg, Germany; schmitter_m@ukw.de; 2 Faculty I, Nursing Science, Department of Behavioural Medicine and Principles of Human Biology for the Health Sciences, Trier University, Universitätsring 15, 54296 Trier, Germany; stefan.m.schulz@uni-trier.de; 3 Department of Oral and Maxillofacial Plastic Surgery, University Hospital Würzburg, Pleicherwall 2, 97070 Würzburg, Germany; mueller_u2@ukw.de (U.M.-R.); kuebler_a@ukw.de (A.K.); kasper_s@ukw.de (S.K.); hartmann_s2@ukw.de (S.H.)

**Keywords:** frailty, geriatric cancer patient, recurrent/metastatic head and neck squamous cell carcinoma, oral health-related quality of life, prosthetic rehabilitation, oral functional capacity

## Abstract

To define frailty in older cancer patients, the aim of this study was to assess the geriatric status and quality of life (QoL) aspects in patients suffering from recurrent/metastatic head and neck squamous cell carcinoma (r/m HNSCC) under palliative treatment. A comprehensive geriatric assessment (CGA) was performed on 21 r/m HNSCC patients at two defined assessments, and the QoL aspects and the impact of descriptive data were evaluated. The Kolmogorov–Smirnov test, Spearman’s rho correlation, and two-way mixed ANOVA were used for statistical analysis. All patients were found to be “frail”. Pain, fatigue, and the burden of illness were the highest-rated symptoms. Oral function and orofacial appearance were highly impaired. A significant impact of descriptive data on the CGA and QoL results was found (all *p* ≤ 0.05). Thus, the CGA results revealed high frailty, severe comorbidities, and high impairments in QoL aspects. The CGA and QoL results were negatively affected by the primary HNSCC treatment approach, the need for prosthetic treatment, and worse oral functional capacity. Therefore, frailty in r/m HNSCC patients seems to be multidimensional. The evaluation of the CGA and QoL aspects in r/m HNSCC patients can be recommended to detect special needs, organize aftercare, and improve the support for frail and vulnerable cancer patients to create a multidisciplinary treatment approach.

## 1. Introduction

Worldwide, head and neck cancer (HNC) represents one of the most common cancer entities [1]. Ninety percent of HNC is squamous cell carcinoma of the head and neck (HNSCC), with increasing incidence and high prevalence (especially in patients of 65 years and older) [2,3]. In addition to extrinsic risk factors, like consumption of tobacco products and alcohol, viral infections (predominantly human papillomavirus), and genetic and epidemiological aspects represent the risk factors for developing HNSCC [4,5,6].

The established treatment regime typically involves surgery, radiotherapy, and chemotherapy, with the modality of intervention depending on the histopathological staging and location of the primary cancer [7]. Despite evidence-based and multimodal treatments, HNSCC shows a high risk of recurrence and metastases, worsening the prognosis for patients [8,9,10].

For the treatment of recurrent or metastatic HNSCC (r/m HNSCC) disease, systemic and palliative therapy is indicated in patients who are not amenable to local invasive treatment of the recurrence by resection (“salvage surgery”) and/or radiation [6,11]. Here, the application of immune checkpoint inhibitors (Pembrolizumab, Nivolumab) or monoclonal antibodies (Cetuximab) is approved for first- and second-line treatment [6,12,13]. Treatment by Nivolumab and Pembrolizumab is based on the interactions with programmed cell death 1 ligand (PD-L1) [14,15], leading to an activation of the T-cells and an antitumor immune response [16]. As described in the KEYNOTE-048 trial, Pembrolizumab is the first treatment option for the vast majority of patients since approximately 85% of the HNSCC tumors are PD-L1 positive [17]. Cetuximab represents an IgG1 monoclonal antibody against the ligand-binding domain of the abnormally activated and overexpressed epidermal growth factor receptor (EGFR) [18], thus down-regulating tumor growth and improving the cytotoxic effect of radiation [12,18]. Based on these findings, the EXTREME trial by Vermorken et al. has set the Cetuximab-based standard for the treatment of r/m HNSCC patients for more than a decade [19].

In general, classical HPV-negative HNSCC mainly occurs in patients with a mean age of 60 to 70 years, and the development of a recurrence is often described within two years after treatment [9,20,21]. Due to the demographic change in the global population, the number and age of HNSCC patients will continue to rise in the next years [20]. Thus, when treating older HNC patients, geriatric aspects, such as comorbidities and frailty, have to be considered regularly [20]. A comprehensive Geriatric Assessment (CGA) using multidimensional assessment tools is helpful for identifying frail patients and guiding clinicians’ treatment decisions [22,23,24]. Questionnaires allowing for an additional evaluation of the patient’s psychological impairments and quality of life (QoL) aspects (health-related quality of life (HRQoL), oral health-related quality of life (OHRQoL)) complement this geriatric assessment [23]. Nowadays, QoL aspects have become mandatory parameters in clinical studies and demonstrate the superiority of immunotherapy compared to classical chemotherapy in recent publications [25].

With regard to the fact that predominantly older patients develop (recurrent) HNSCC, it also has to be considered that they are already suffering from the consequences of the initial HNSCC therapy, such as impaired oral functions (chewing, swallowing, tasting), plus the high prevalence of malnutrition in patients after HNSCC therapy [26,27]. In addition to these physical impairments, cancer patients often suffer from mental health problems, such as anxiety and depression [28,29]. This can significantly reduce patients’ overall QoL and OHRQoL [3,30], adding to impaired CGA results and higher frailty.

To increase patients’ QoL and OHRQoL, interdisciplinary support and special after-care of HNSCC patients is of major importance. In this line of treatment, oral rehabilitation can also have a positive impact on oral functions and psychological well-being [30,31]. In addition to the loss of hard and soft tissue, limited mouth opening, xerostomia, and other limiting factors after tumor resection, adjuvant radiation, and chemotherapy, the oral health and treatment options of older patients are influenced by their geriatric status. Thus, when planning the individual dental treatment of frail older patients, the oral functional capacity (OFC) has to be considered. The OFC represents a dental geriatric assessment tool and can classify patients into four resilience capacity levels, which helps to evaluate the prosthetic treatment options [32,33].

However, the performance and evaluation of CGA and OFC are time-consuming and are not routinely performed in clinical practice. Furthermore, older patients with recurrent HNSCC are strongly underrepresented in previous studies, indicating a demand for further investigation [20].

To identify the geriatric status and special needs of recurrent/metastatic HNSCC patients, the aim of this study is to monitor geriatric parameters, QoL aspects, and options for prosthetic rehabilitation during palliative treatment. Furthermore, the impact of the descriptive parameters on geriatric aspects was evaluated.

The following null hypotheses were formulated:

(I) The GCA results, psychological parameters, and HQoL/OHRQoL aspects are not impaired in patients with recurrent/metastatic HNSCC.
(II) The primary therapy of HNSCC, oral functional capacity, patient age, and the need for prosthetic rehabilitation show no impact on the subjective and objective CGA parameters.

## 2. Materials and Methods

### 2.1. Study Design

This study was approved by the Ethics Committee of the University School of Medicine (approval number: 25/22). This study was conducted from May 2022 to January 2023.

Patients who developed metastasis or recurrence of HNSCC after completing treatment for primary HNSCC and attended appointments for palliative treatment at the Department of Oral and Maxillofacial Plastic Surgery at a German university hospital were included. Patients had to be aged 18 years or older. Exclusion criterion was inability to complete informed consent. Thus, written informed consent was obtained from all patients who agreed to participate.

A comprehensive geriatric assessment (CGA) was performed, and patients completed standardized questionnaires at two appointments of their treatment course within 3 months (T1: first assessment, T2: second assessment after an interval of 3 months of treatment). In addition, descriptive data and oral functional capacity were assessed.

### 2.2. Comprehensive Geriatric Assessment (CGA)

#### 2.2.1. Objective Geriatric Assessment

Table 1 presents the objective CGA tools and how they were performed to evaluate geriatric parameters.

#### 2.2.2. Questionnaires

In addition to objective CGA, self-assessment of frailty parameters was evaluated with psychological parameters, HQoL, and OHRQoL. To measure OHRQoL, all patients completed the German version of the Liverpool Oral Rehabilitation Questionnaire version 3 (LORQv3), consisting of 40 items rated on a 4-point Likert scale [30,53]. The first section contains 17 items related to oral function (OF), orofacial appearance (OFA), and social interaction (SI). The second section has 23 items evaluating the respondents’ problems and satisfaction with their dentures/implants. A higher LORQv3 score indicates poorer OHRQoL.

The Hospital Anxiety and Depression Scale (HADS) was used to measure psychological impairment. The questionnaire consists of two subscales related to anxiety (HADS-A) and depression (HADS-D), consisting of 7 items each [54]. Items were scored with 0 to 3 points, with a maximum of 21 points in each subscale. The cut-off value for likely presence of clinical levels of anxiety and depression has been defined as ≥8 points, respectively [55].

To assess HQoL, patients answered the European Organisation for Research and Treatment of Cancer (EORTC) Core QoL Questionnaire version 3 (QLQ-C30) and the EORTC elderly cancer patients module (ELD-14) [56].

In the QLQ-C30, 28 questions cover a symptom scale (fatigue, nausea and vomiting, pain, dyspnoea, insomnia, appetite loss, constipation, diarrhea, and financial difficulties), and a functional scale (physical, role, emotional, cognitive, and social functioning). Items are rated from 1 (not at all) to 4 (very much). In addition, 2 items can be answered on a 7-point scale, which evaluates the global health status and overall QoL [57,58]. A higher score represents higher QoL, function, and symptoms [59].

The ELD-14 questionnaire is a complement to the QLQ-C30 and asks for age-specific items of major importance in older cancer patients [60,61]. Items were also divided into a symptom and a functional scale. Functional items are related to maintaining purpose and family support. Symptom scales include mobility, worries about others, future worries, burden of illness, and joint stiffness. According to recommendations of the EORTC, raw scores were transformed linearly into a score from 0 to 100 [58].

#### 2.2.3. Frail Grouping

According to previous studies, patients without any restrictions in CGA parameters G8, MMST, Barthel Index, IADL, MNA, CCI, and TUG, were defined as “normal function” patients who had restrictions in one of these CGA parameters were defined as “pre-frail” and patients who reached the cut-off value in at least two parameters were defined as “frail” [45,49,62].

### 2.3. Descriptive Data and Oral Functional Capacity

The following parameters were collected, and groups were built:

(I) Age: <65 years, ≥65 years.
(II) Need for prosthetic treatment: yes; no.
(III) Oral functional capacity: The OFC was classified depending on the following parameters: therapeutic capability, oral hygiene ability, and self-responsibility of the patient. OFC was classified into four resilience capacity levels [32,33]. From these parameters, the criteria with the lowest grade were used to specify the resilience capacity level (RCL) as follows [63]:
  RCL1: Normal.
  RCL2: Slightly reduced.
  RCL3: Greatly reduced.
  RCL4: No resilience.
(IV) Type of primary HNSCC treatment completed: surgery and radiotherapy ± chemotherapy; and surgery only.

### 2.4. Statistical Analysis

The Kolmogorov–Smirnov test was used to test for normal distribution. The impact of the parameters ‘patients age (≥/< 65 years), need for prosthetic treatment, oral functional capacity, age, and primary HNSCC therapy on CGA parameters and questionnaires were determined using linear regression models.

Spearman’s rho correlation (*r_s_*) was used to investigate correlations between CGA parameters and questionnaire subscales and was interpreted in line with established criteria (*r_s_* = 0.1 poor, *r_s_* = 0.3 moderate, *r_s_* = 0.5 = strong) [64].

Two-way mixed ANOVA was performed to determine main effects of assessment time (T1–T2). Mauchley’s test was used to test for sphericity, Greenhouse Geisser correction was applied in case of lack of sphericity. Partial eta-squared (*η^2^*) was added as a measure of effect size, with 0.01, 0.06, and 0.14 reflecting small, medium, and large effects, respectively. Missing data at T2 assessment were imputed using the conservative last observation carried forward approach. In a sensitivity analysis, we compared this to results obtained with available data only. Of note, the results remained essentially the same. Details of this analysis are available upon request from the corresponding author. Data were analyzed with SPSS version 28.0 (SPSS, Chicago, IL, USA) and the default level of significance was set at α ≤ 0.05.

## 3. Results

### 3.1. Study Population and CGA Parameters

In total, 21 patients with a mean ± SD age of 70.46 ± 10.94 years participated in this study (female: 7, male: 14). Within this study period, one patient died and three patients developed further severe (neuronal) health problems and changed from a stable disease condition into an unstable, insecure condition. The drugs applied for immunotherapy were Nivolumab in 4 patients, Pembrolizumab in 12 patients, and Cetuximab in 5 patients. The descriptive parameters and r/m HNSCC-related information of the patients (UICC stadium, primary HNSCC treatment) are shown in Table 2. The patients’ final TNM stadium is presented in Supplementary Table S6.

### 3.2. CGA Assessment

**Figure 1:**
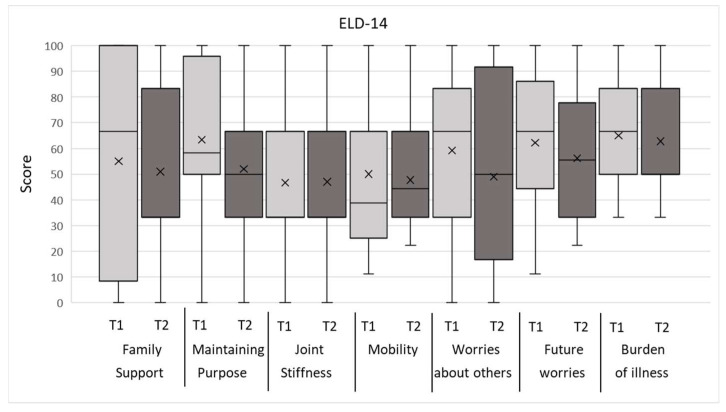
Box plots for EORTC-QLQ-ELD-14 questionnaire items at T1 and T2 assessments; see text for details.

**Figure 2:**
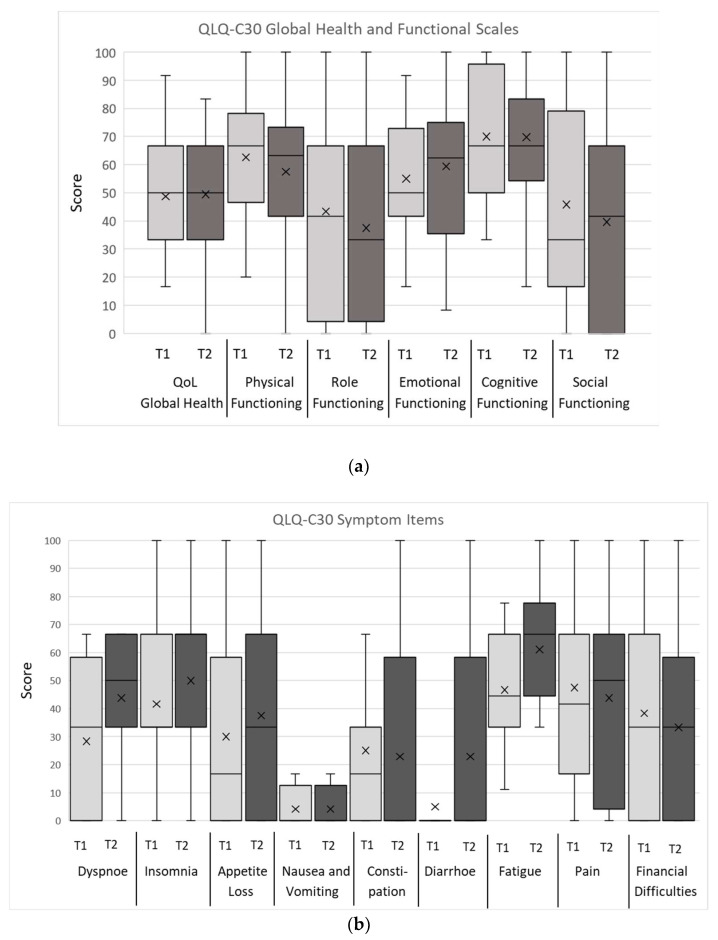
(**a**) Mean values of EORTC-QLQ-C30 questionnaire Global Health and Functional Items, at T1 and T2 assessments; see text for details. (**b**) Box plots for EORTC-QLQ-C30 questionnaire Symptom Items, of the entire patient population at both assessment times (T1/T2); see text for details.

**Figure 3:**
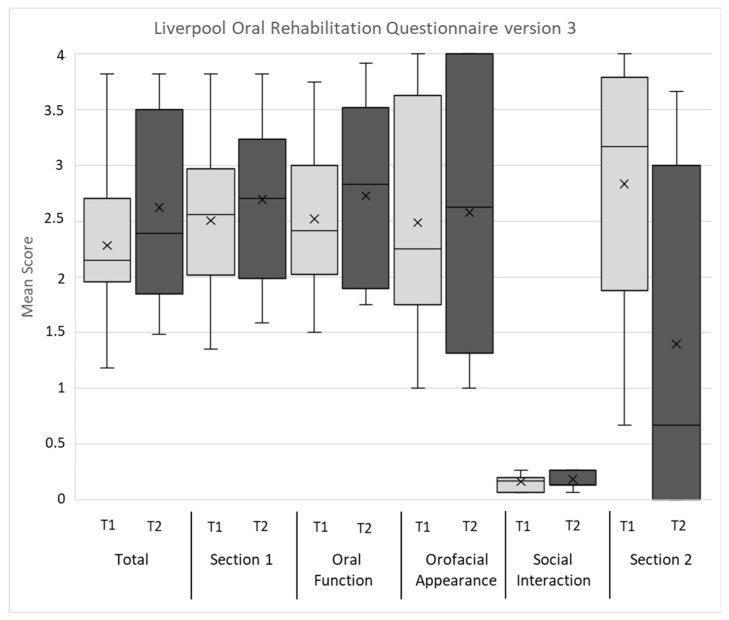
Means of Liverpool Oral Rehabilitation Questionnaire version 3 results at first (T1) and second (T2) assessments; see text for details.

Table 3 and Table 4 present the overall results of the objective CGA parameters. The evaluations of the QoL/OHRQoL and psychological aspects by the questionnaires, QLQ-C30, ELD-14, LORQv3, and HADS, of the entire patient population are shown in Figure 1, Figure 2 and Figure 3, with the mean marked by X and the middle line of the box, respectively. The upper and lower limits of the box cover the interquartile range, with the boundaries matching the 25% and 75% quartile, respectively. No outliers or extreme scores were present. The whiskers show the minimum and maximum values.

CCI was mainly affected by the presence of a tumor and the recurrence or metastasis of head and neck squamous cell carcinoma. In addition, myocardial diseases (*n* = 4), vascular diagnoses (peripheral vascular disease and cerebral vascular accidents (*n* = 6)), dementia (*n* = 1), internistic diagnoses (pulmonary disease (*n* = 2), connective tissue disorder (*n* = 1), liver disease (*n* = 2), diabetes and its complications (*n* = 4), and renal disease (*n* = 6) occurred. Moreover, one patient suffered from AIDS.

Results from the QLQ-C30 revealed that the symptoms of pain and fatigue were rated highest and role and social functioning lowest. In the ELD-14, the burden of illness was the symptom scale rated the highest. The LORQv3 results indicated the highest impairments in the domains of oral function and orofacial appearance. The HADS evaluations of depression were higher than anxiety. All the patients reached the cut-off value in at least two CGA parameters at both assessment times and can, therefore, be defined as “frail”. The detailed explorative data of the mean values according to the regression variables of the primary HNSCC therapy, oral functional capacity, need for prosthetic treatment, and age at the first and second assessments are presented in Supplementary Tables S1–S5.

### 3.3. Regression Analysis

The regression analysis showed significant effects for the HNSCC therapy, oral functional capacity, patients age (≥/<65 years), and need for prosthetic treatment on the assessed CGA parameters (all *p* ≤ 0.05, β ≥ −0.429, R^2^ ≥ 0.184). No significant effect of any of these predictors on the CGA parameters, CCI, TUG, ADL, and HADS-A questionnaire, emerged (all *p* ≥ 0.052, β ≤ 0.429, R^2^ ≥ 0.182).

Depending on the oral functional capacity, significant results at both assessment times demonstrated increasing evaluations of the questionnaires, LORQv3, ELD-14, and HADS-D, reflecting increasing oral functional capacity. Negative correlation coefficients demonstrated higher impairments of the G8, IADL, and MNA, which are descending in the following order of OFC: 3 > 4 > 2 > 1 (all *p* ≤ 0.05, β ≥ −0.482, R^2^ ≥ 0.232) (Table 5).

The regression analysis of the primary HNSCC therapy indicated a higher MMST score, QLQ-30, and ELD-14 evaluations with radiotherapy ± chemotherapy (all *p* ≤ 0.05, β ≥ 0.464, R^2^ ≥ 0.215) (Table 5).

The parameter age had a significant impact on LORQv3 orofacial appearance at the T1 assessment (*p* ≤ 0.03, β = −0.541, R^2^ = 0.293). A negative correlation indicated decreasing OFA evaluations in patients ≥ 65 years.

Patients with the need for prosthetic treatment had lower global health and higher LORQv3 scores (all *p* ≤ 0.48, β ≥ −0.447, R^2^ ≥ 0.200) (Table 5).

### 3.4. Correlation Analysis and Effect across Time

Spearman’s rank correlation revealed significant associations between several objective CGA parameters and subjective questionnaire evaluations of the ELD-14 and QLQ-C30 at the T1 and T2 assessment times (all *p* < 0.05) (Table 6).In significant correlations at T1, the correlation coefficient ranged between *r_s_* = *−*0.45 and *r_s_* = 0.61, indicating moderate to strong correlations. The T2 assessment demonstrated high correlations between *r_s_* = −0.49 and *r_s_* = 0.78, suggesting strong correlations. The details of this analysis are presented in Supplementary Tables S7 and S8.

The changes across time were mainly not significant (F (1,20) ≤ 4.174, *p* ≥ 0.055, partial-*η*^2^ ≤ 0.180). The significant effects across time were only found in the IADL (F (1,20) = 6.968, *p* = 0.016, partial-*η*^2^ = 0.258), indicating a significant decrease from T1 to T2.

## 4. Discussion

The aim of this study was to assess the geriatric status, quality of life aspects, and the status and ability for prosthetic rehabilitation in patients with recurrent/metastatic HNSCC under palliative treatment. The present results demonstrated high impairments among patients suffering from r/m HNSCC in the comprehensive geriatric assessment results, psychological parameters, and HQoL/OHRQoL aspects, and the first null hypothesis can, therefore, be rejected. Thus, all the patients in this present study reached the cut-off value in at least two CGA parameters and can be defined as “frail” at both assessment times [45,49]. The presented patient cohort was characterized by a need for palliative treatment. However, the TNM and UICC classifications were sometimes misleading since those parameters did not reflect the patients’ history. Fifteen out of twenty-one patients were classified as UICC IV, but the remaining patients were treated with systemic therapy based on a lower UICC stage. This was due to missing alternative options in those heavily pretreated patients. One patient, for example, developed his sixth carcinoma after multiple surgeries and adjuvant radio-chemotherapies and was finally only suitable for immunotherapy. Notably, our study did not target oncologic treatment outcomes but focused on geriatric and quality of life aspects. Therefore, our patient sample was homogeneous with regards to being not suitable for surgery or radiotherapy in certified high-throughput cancer center with long-lasting experience in oncologic surgery and reconstruction. Thus, the high number of treatments impaired the outcome of the patients and may have contributed to the high frailty in patients suffering from r/m HNSCC.

### 4.1. CGA and QoL Impairments in Patients with r/m HNSCC Were Higher Than after Primary HNC Therapy

As all the patients reached the cut-off value of the G8 for being “frail” at the T1 and T2 assessments, the number of abnormal G8 values was higher than in HNSCC patients with primary disease without recurrence or metastases [34]. This is consistent with previous findings that have also demonstrated abnormal G8 values ≤ 14 points in 100% of their patients suffering from r/m HNSCC [65]. Therefore, a full CGA is useful in identifying special needs in the present patient population suffering from recurrent/metastatic HNSCC.

In addition, restrictions in the Barthel Index and Instrumental Activities of Daily Living were observed in at least 13 patients at T1 and 14 patients at the T2 assessment, demonstrating the impairment of daily activities in our patients. This is in accordance with Silver et al., who explained the impairments of IADL and ADL in HNSCC patients by difficulties in chewing or swallowing, weight loss, fatigue, and perceived stress [66]. The present results confirm these findings that patients with higher difficulties in oral functions (need for prosthetic treatment, RCL 3) were characterized by lower ADL and IADL values than patients without the need for dental prosthetic treatment or a lower RCL. However, the present results also revealed a decrease in IADL and the Barthel Index evaluation between the T1 and T2 assessments. This can be explained by three patients needing additional medical intervention and in-patient treatment during the course of this study.

The difficulties in chewing and swallowing also affected the nutritional status of the patients. The present MNA results indicated an impaired nutritional status. Despite one patient at the T2 evaluation, the majority of patients demonstrated deficits and were classified as either “malnourished” (T1: 43%, T2: 40%) or “at risk for malnutrition” (T1: 57%, T2: 55%) [34,51]. Previous findings by Dewansingh et al. demonstrated a coexistence of malnutrition and frailty in 21% of HNC patients, which led to a prevalence of a malnutrition risk of 39% in frail patients [67]. Although seven patients in our sample even had a Percutaneous Endoscopic Gastrostomy in order to improve the nutritional outcome of cancer patients, and weight control was applied during treatment, the nutritional status of the r/m HNSCC patients seems to be more impaired compared to patients suffering from primary HNC as described by Kramer et al. [27]. Thus, an increased awareness of frailty can be expected to have a positive impact on nutritional status.

Furthermore, malnutrition and frailty are significantly associated with comorbidity [27], a finding that is also consistent with the present results. A total of 71.4% (T1) and 70% (T2) of the patients in the current sample had CCI grade three, indicating severe comorbidities. This is higher than in previous studies on HNC patients, which have mainly included patients without recurrence/metastatic disease [21,23,68]. Although there is disagreement about whether comorbidities affect the treatment approach in HNC/HNSCC patients, previous findings have demonstrated that mortality and the prognosis of HNC and HNSCC patients are negatively influenced by existing comorbidities [69,70,71,72]. In our patients, severe comorbidities were highly prevalent. This may be explained by their higher age when developing recurrence/metastatic cancer disease, the presence of tumor disease, the development of metastasis, and adverse side effects of the primary cancer therapy compared to other studies [69,70,71,72]. Noteworthy, the ineligibility for salvage surgery or (re-)irradiation is oftentimes based on prevalent comorbidities and frailty. As a result, patients in r/m situations are often subjected to palliative drug treatment. This might lead to bias in findings regarding the examined cohort. However, this only further underlines that knowledge about existing comorbidities, especially in patients with recurrent or metastatic cancer disease, is of major importance.

The effects of the primary HNSCC therapy were also reflected in the subjective QoL evaluations and the psychological status of this present study population. The present QLQ-C30 results of symptom, functional, and global health aspects indicated high impairments in HQoL, which is in line with findings in previous studies [73,74]. The highest-rated symptoms reported were fatigue and pain, indicating a high burden of symptoms. Role and social functioning were the lowest evaluated functions, indicating high impairment in daily and social activities. This is consistent with the impairments present in the ADL/IADL results, as well as previous findings in patients suffering from cancer in general and recurrent/metastatic HNSCC [73,74]. In addition, Aghajanzadeh et al. and Kramer et al. demonstrated impairment in the functional, symptomatic, and global health scales in HNC patients after primary therapy, which, however, was less severe than in our patients suffering from r/m HNSCC [27,75]. In addition to a worse global QoL in patients with primary HNC compared to patients suffering from other malignancies, the global health values of this present study sample were also worse than in previously examined samples [23,27,75]. This may be explained by the (long-term) side effects of primary HNSCC therapy, the development of recurrence or metastatic HNSCC, and the high frailty in our patient population. Consistent with this perspective, we found that the effects of the patients’ physical condition on family life and social activities, as well as limitations in daily activities, were associated with lower values in patients with more impaired oral function (i.e., in the group “RCL 3”, requiring prosthetic treatment). This indicates higher social restrictions in patients with higher physical impairment, which is also reflected in the objective CGA parameters. Worse evaluations in the G8, ADL, IADL, and MNA of patients, with higher impairments in oral function, also suggested high frailty, associated with restrictions in HQoL. This is in accordance with previous findings, where an association between frailty and the HQoL of cancer patients has been demonstrated already [76]. Notably, changes between the T1 and T2 assessments were mainly not clinically meaningful, suggesting a stabilization of QoL under therapy. The stabilization of functional, symptom, and global health levels in patients suffering from recurrent/metastatic HNSCC under treatment with Nivolumab, Pembrolizumab, and Cetuximab were already described previously [73,77]. A stabilized QoL could be explained by regular attendance to treatment appointments, providing a regular and standardized supply of necessary rehabilitation measures and medication, possible psychosocial support associated when interacting with medical caregivers, and psycho-oncologic support (see below). Nonetheless, implementing CGA could provide valuable insight for the implementation of further enhancements for individualized and appropriate patient support.

The ELD-14 questionnaire was used for detecting specific needs and the HRQoL aspects in elderly cancer patients. The present results demonstrated the highest impairment in the burden of illness domain, indicating a high burden due to the cancer disease, which is in line with previous reports [74]. In this present study population, a high symptom burden correlated with high frailty and comorbidity, which are associated with the adverse side effects of the primary therapy, restricted oral functions, and impaired oral rehabilitation ability [78]. In the present results, this is supported by the highest burden in patients with the need for prosthetic treatment and RCL 4. In line with this, a high rating in the burden of illness domain due to adjuvant radiotherapy, the side effects of HNC treatment, and high frailty due to age in elderly cancer patients have been described previously [74,79,80].

Moreover, the negative effects on HRQoL were associated with a reduced OHRQoL. Using the LORQv3, high impairments were found for the domains of oral function and orofacial appearance, indicating a low ability of, i.e., chewing, swallowing, and mouth opening. In addition to the side effects of surgical treatment and radio-/chemotherapy (xerostomia, restricted agility of oral structures, and facial disfigurement), the development of a recurrence/metastatic disease seems to cause an additional deterioration of OHRQoL compared to patients with primary HNC disease [26,30,81]. An additional negative effect on OHRQoL was found in the “need for prosthetic treatment”. The high incidence of frailty in patients suffering from r/m HNSCC has a negative impact on the ability for oral rehabilitation, as frailty, and coexistent factors, such as comorbidity and malnutrition, can lead to wound healing disorders, psychological problems, and restricted surgical and prosthetic treatment abilities [33,67,82]. The evaluation of the LORQv3 domain of oral function also revealed swallowing problems, which were described as a predictor for physical fatigue [83]. This is reflected in our, and previous QLQ-C30, results with high fatigue in r/m HNSCC patients, which underlines the association between physical and HRQoL/OHRQoL impairment [84]. These limitations also affected the psychological parameters, as our patients demonstrated a higher psychological burden than scores reported for the general population of Germany [85].

Depression was rated higher than anxiety, which is in line with a similar effect present in the population values and descriptions of other HNSCC patients [83,85]. The highest anxiety was described at the time of diagnosis, and depression was highest 3 months after the beginning of the treatment. Both decreased in the further treatment process of the primary HNSCC therapy [83]. According to Singh et al., patients suffering from r/m HNSCC reported moderate or extreme depression/anxiety, which, however, was not measured using the HADS scales [86]. Furthermore, a high level of psychological distress was already observed previously in HNC patients [29]. However, the anxiety and depression level of our patients was lower than in previous investigations [83,85,86]. This could be explained by the psycho-oncologic support of our patients at the time of diagnosis and, if required, during treatment.

Thus, the impairments in the HRQoL/OHRQoL and psychological parameters reflect the high frailty in patients suffering from r/m HNSCC measured using objective CGA criteria. This observation is also reflected in the present correlation results, where moderate to high correlations indicate a high consistency of self-reported (questionnaires) and objectively (test performances) assessed CGA results.

In conclusion, when comparing the present results with previous studies, the restrictions of patients suffering from r/m HNSCC were higher than in primary HNC/HNSCC patients. Therefore, the evaluation of geriatric parameters is recommended for optimal treatment planning.

### 4.2. Primary HNSCC Treatment, the Need for Dental Prosthetic Treatment, and Worse Oral Functional Capacity Negatively Affected CGA Parameters, HQoL, and OHRQoL

Furthermore, the influence of the descriptive parameters on CGA was evaluated, and influencing factors were found. Therefore, also the second part of the null hypothesis can be rejected.

Regarding oral functional capacity, our patients suffering from r/m HNSCC predominantly demonstrated RCL3, which implies greatly reduced therapeutic capability and oral hygiene ability, as well as reduced self-responsibility. This is comparable to frail elderly dental patients in Germany (≥75 years) with a care level [32,33]. This suggests that our comparatively “younger” sample (mean age 70.46 ± 10.94 years) was also characterized by high impairments in the dental geriatric assessment tool, indicating a negative effect of frailty on dental treatment ability, and the dental treatment of frail older patients is complex [87]. This is reflected in the present results. All the patients were identified as “frail”. Thus, the present results indicated a significant association of RCL with “frailty parameters”, which were associated with stronger impairment shown in the G8, ECOG, IADL, and MNA scores, based on the patients’ self-responsibility, therapeutic capability, and oral hygiene ability. In addition, the nutritional status of the HNC patients was influenced by the ability for oral function mastication and mouth opening. The latter has also been shown to affect RCL [33]. Thus, higher RCL indicated higher restrictions in the CGA parameters, as well as a higher frailty risk in our patient sample suffering from r/m HNSCC. Moreover, HRQoL and OHRQoL were significantly affected by RCL (LORQv3, HADS-D, ELD-14, QLQ-C30 questionnaires). Here, a higher RCL was associated with higher depression (HADS-D), higher burden of illness and more future worries (ELD-14), lower social functioning (QLQ-C30), and lower OHRQoL aspects, such as oral function, orofacial appearance, and social interaction (LORQv3). Thus, the impaired ability of oral functions and reduced resilience capacity levels due to oral cancer effects and treatment side effects correlated with the negative social aspects, which additionally worsens the burden due to r/m HNSCC. Despite psychological support, the prosthetic rehabilitation of HNC patients can significantly improve OHRQoL and should be part of an interdisciplinary treatment concept [30,81,88]. This is in line with our findings, where the need for prosthetic treatment also was associated with a significant increase in scores on the global health scale (QLQ-C30). However, it should be noted, that the highly impaired RCL restricts abilities for dental prosthetic rehabilitation in r/m HNSCC patients.

In addition to OFC, the primary HNSCC treatment also demonstrated significant effects on the fatigue subscale of the QLQ-C30, with adjuvant radio-/chemotherapy leading to higher levels of fatigue. Thus, fatigue represents a common problem in patients suffering from HNSCC. Berg et al. have described a significant increase in fatigue in patients with HNC from diagnosis to 1 year under treatment, and an improvement to baseline levels after 2 years [83]. It should be noted that the development of a recurrence often has been described within the first two years after primary treatment. Hence, this patient population can hardly recover from the first treatment approach, and fatigue should be recognized as a major burden in r/m HNSCC patients [9,20,21]. In addition, the high frailty, low HQoL/OHRQoL, and deteriorated psychological status in these patients are likely explanations for the high prevalence of fatigue. Unsurprisingly, fatigue in HNC/HNSCC patients was significantly associated with HADS-D/A above cut-offs, indicating the likely presence of clinically relevant psychological burden, local pain, swallowing problems, low global health score, high ECOG, and low weight in previous investigations [83,89].

Interestingly, there was no significant association between the patients’ age and objective CGA parameters, QLQ-C30, ELD-14, and HADS results. In this present study, groups were built depending on whether the age of ≥65 years was reached or not. Thus, patients aged ≥65 years belonged to the group of “elderly” patients, which is a common standard in line with previous research [90]. However, the present results indicate no impact of age on the frailty parameters, and a high incidence of frailty was found even among “younger” patients <65 years. This underlines the need for evaluating geriatric aspects of patients suffering from r/m HNSCC in all patients, not only in elderly patients. However, patients’ age could be considered as a limitation as some CGA tools are validated only for patients >65 years. Hence, validation for further age groups appears advisable. Nevertheless, previous CGA investigations have also included patients younger than 65 years [91]. The current results could also be influenced by the small sample size, which has to be mentioned as a limitation of this current study. However, the small sample size is typical for a homogeneous patient sample suffering from r/m HNSCC and treated with immunotherapy, as demonstrated by previous investigations on a similar topic [65]. Nevertheless, the distribution of age and gender in this present study population is typical for HNSCC patients and the cohort can, therefore, be considered representative [92]. However, due to the limited sample size, dimorphic differences within the CGA results were not investigated, which could be addressed in further studies with a larger patient sample. Another limitation is the comparison to other HNC studies in general. To the best of the authors’ knowledge, the number of CGA studies in HNSCC patients and r/m HNSCC is quite limited. However, HNSCC represents the most common HNC and, hence, could be considered the “second best” available choice for comparisons. An additional limitation is that the T1 assessment was not performed at a defined time of treatment application, and the impact of the treatment cycles could, therefore, not be investigated. Regardless of these limitations, the performed CGA in this study was based on a multidimensional assessment within medical, psychological, social, and functional domains, and, therefore, contained essential key points of the CGA with high informative value [93].

## 5. Conclusions

Based on our findings, the following conclusions can be drawn:

(I) The comprehensive geriatric assessment revealed high frailty, severe comorbidities, and high impairments in the QoL aspects in patients suffering from recurrent/metastatic HNSCC under palliative treatment. The symptom items of pain, fatigue, and the burden of illness were rated highest. In addition, oral functions and orofacial appearance were highly impaired. Missing effects across time indicated a stabilization of the QoL parameters under treatment.
(II) The primary multimodal HNSCC treatment approach with surgery and radiotherapy ± chemotherapy, the need for dental prosthetic treatment, and worse oral functional capacity negatively affected the CGA parameters, HQoL, and OHRQoL.
(III) The present results indicated that the high frailty in r/m HNSCC patients was affected by multidimensional aspects, influencing the geriatric parameters, QoL aspects, and descriptive data, which can be assessed using CGA and standardized questionnaires.
(IV) For detecting special needs, organizing aftercare, and improving support for frail and vulnerable cancer patients, a comprehensive assessment of the geriatric status and QoL aspects can be recommended when the cut-off value in the G8 Screening Tool is reached. This could be implemented easily in the everyday clinical treatment of these patients.
(V) Although high impairments in patients suffering from r/m HNSCC will usually not fully recover, appropriate aftercare can help to improve the patients’ outcomes both medically and psychosocially. Therefore, a multidisciplinary treatment approach, including nutritional interventions, psycho-oncological support, and dental prosthetic treatment, can be recommended. As chewing, swallowing, dental rehabilitation, and orofacial reconstruction are complex domains that are crucial for patients’ quality of life, the involvement of specialists with expertise in dental care, maxillofacial surgery, and ear, nose, and throat in the interdisciplinary treatment team is considered mandatory.

## Figures and Tables

**Table 1:** Overview of objective comprehensive geriatric assessment tools.

Assessment Tool	Performance and Additional Information
G8 Screening Tool (G8) [34,35,36]	G8 was used as a geriatric screening instrument to identify the geriatric risk profile of patients, which includes items related to food intake, weight, mobility, neuropsychological status, drug intake, and age. The maximum total score is 17, with a cut-off value of ≤14 points.
Charlson Comorbidity Index (CCI) [37,38,39,40]	To classify comorbidities that accompany cancer, the recently used CCI version includes 19 medical conditions, which can significantly influence the patient’s overall survival. For classification, the ICD-10 versions by Glasheen et al. were used. These conditions are weighted according to the relative mortality risk. The total score of all weighted conditions is used to calculate the comorbidity index for classifying the comorbidity grade: 0: No comorbidity (comorbidity index: 0) 1: Mild comorbidity (comorbidity index: 1–2) 2: Moderate comorbidity (comorbidity index: 3–4) 3: Severe comorbidity (comorbidity index: 5)
Eastern Cooperative Oncology Group Performance Status (ECOG) [41]	The activity status is classified into 6 grades from unrestricted activity (ECOG grade 0) to death (ECOG grade 5).
Timed up and Go Test (TUG) [42,43]	TUG was conducted to assess the fall risk. Time needed by patients to get up into standing position from an armchair, walk a distance of 3 m there and back, and sit down again on the chair is measured. TUG time > 13.5 s and the inability to perform this task were defined as cut-offs. Whether or not the patient surpassed the cut-off was used for statistical analysis.
Barthel Index of Activities of Daily Living (ADL) and Instrumental Activity of Daily Living (IADL) [44,45,46,47,48,49]	The Barthel Index of ADL, based on the Hamburg Manual, was used to evaluate the performance of activities of daily living. Ten performance items on activity and mobility were rated from 0 (minimum) to 100 (maximum) points. IADL items were based on 8 items of related instruments of daily living, such as telephone usage, shopping, and housekeeping. A higher score indicated higher independence in daily living. Restrictions in daily living were defined as a score of <100 points for ADL and ≤7 points for IADL.
Mini Nutritional Assessment (MNA) [34,50,51]	The MNA was used to assess malnutrition by evaluating 18 questions concerning anthropometric, global, dietetic, and subjective sections. A sum score of a maximum of 30 points was computed. Scores were classified into: ≥24: well-nourished, 17–23.5: risk of malnutrition, and <17: malnourished. A score ≤23.5 points was, therefore, considered abnormal.
Mini Mental State Examination (MMSE) [49,52]	The MMSE was applied to measure cognitive function related to orientation, memory, attention, and language, with a maximum score of 30 points. The cut-off for a decline in cognitive function was defined as ≤23 points.

**Table 2:** Descriptive data of all participants.

Parameter	Variables	T1 First Assessment	T2 Follow-Up Assessment after 3 Months of Treatment
Sample size	Total n	21	20
Age (years)	≥65	12	11
	<65	8	8
UICC stadium	I	1	1
	II	3	3
	III	2	2
	IV	15	14
Need for prosthetic treatment	yes	18	17
	no	3	3
Oral functional capacity	RCL 1	0	3
	RCL 2	5	2
	RCL 3	15	14
	RCL 4	1	1
Primary HNSCC treatment	surgery and radiotherapy ± chemotherapy	17	16
	surgery only	4	4

RCL: resilience capacity level.

**Table 3:** Results of CGA parameters at first (T1) and follow-up (T2) assessments.

Parameter	Classification	T1	T2	Change T1–T2
CCI (grade)	0	0	0	-
	1	3	2	−1
	2	3	4	+1
	3	15	14	−1
ECOG (grade)	0	1	0	−1
	1	2	5	+3
	2	12	8	−4
	3	6	4	−2
	4	0	3	+3
	5	0	1	+1
TUG	cut-off not reached	14	12	−2
	cut-off reached	7	8	+1

CCI: Charlson Comorbidity Index. ECOG: Eastern Cooperative Oncology Group Performance Status. TUG: Timed up and Go Test.

**Table 4:** Means and standard deviations (SD) of evaluated CGA parameters of the entire patient population at first (T1) and follow-up (T2) assessments.

Assessment Time	T1		T2		Change T1–T2	
CGA Parameter	Mean	SD	Mean	SD	Mean	SD
G8	9.64	2.53	9.28	3.12	0.36	0.59
ADL	91.19	13.41	53.30	17.34	37.89	3.93
IADL	5.86	2.15	4.35	3.31	1.51	1.16
MMST	25.38	4.03	22.20	9.99	3.18	5.96
MNA	17.17	2.91	16.58	5.03	0.59	2.12

G8: G8 Screening Tool. ADL: Activity of Daily Living. IADL: Instrumental Activity of Daily Living. MMST: Mini Mental Status Test. MNA: Mini Nutritional Assessment.

**Table 5:** Significant regression effects of regression predictors, oral functional capacity, primary HNSCC therapy, age, and need for prosthetic treatment, at T1 and T2 assessment times. Item-related scales of the questionnaire scales are presented in italics.

Regression Predictor	T1 CGA Parameter	T2 CGA Parameter
**Oral functional capacity**	ECOG	ECOG
	LORQv3: Section 1*, oral function, orofacial appearance*	LORQv3: Section 1*, Oral Function, orofacial appearance, social interaction* G8 IADL
		MNA HADS-D ELD-14: *Burden of illness, future worries* QLQ-C30: *Insomnia, social functioning*
**Primary HNSCC therapy**	ELD-14: *Family support* QLQ-C30: *Fatigue* MMST	ELD-14: *Family support* QLQ-C30: *Fatigue*
**Age**	LORQv3^:^ *Orofacial appearance*	
**Need for prosthetic treatment**	LORQv3: Section 2 QLQ-C30: *Global health*	

ECOG: Eastern Cooperative Oncology Group Performance Status. LORQv3: Liverpool Oral Rehabilitation Questionnaire version 3. G8: G8 Screening Tool. IADL: Instrumental Activity of Daily Living. MNA: Mini Nutritional Assessment. HADS-D: Hospital Anxiety and Depression Scale- Depression. ELD-14: EORTC-QLQ-ELD14 Questionnaire. QLQ-C30: EORTC-QLQ-C30 Questionnaire. MMST: Mini Mental Status Test.

**Table 6:** Significant Spearman rank correlations between objective CGA parameters and subjective questionnaire evaluations at both assessment times.

Significant correlation between	
Questionnaire and	Objective CGA parameter
EORTC QLQ-ELD-14 Symptom items	CCI grade MMST G8 ECOG ADL IADL MNA
EORTC QLQ-ELD-14 Functional items	CCI grade MMST G8 ECOG ADL IADL MNA
EORTC-QLQ-C30 Symptom items	CCI grade G8 ECOG IADL MNA
EORTC-QLQ-C30 Functional items	CCI grade MMST G8 ECOG IADL MNA

CCI: Charlson Comorbidity Index. MMST: Mini Mental Status Test. G8: G8 Screening Tool. ECOG: Eastern Cooperative Oncology Group Performance Status. ADL: Activity of Daily Living. IADL: Instrumental Activity of Daily Living. MNA: Mini Nutritional Assessment.

## Data Availability

The data presented in this study are available on request from the corresponding author.

## Supplementary Materials

The following supporting information can be downloaded online: https://www.mdpi.com/article/10.3390/jcm12175738/s1. Table S1: the mean values of the OHRQoL measured by LORQv3 (Liverpool Oral Rehabilitation Questionnaire version 3), with regard to the entire patient population and regression predictors at the baseline (T1) and follow-up (T2) assessment; Table S2: the mean values of the psychological questionnaire, HADS, with regard to the entire patient population and regression predictors at the baseline (T1) and follow-up (T2) assessment; Table S3: the mean values of the HQoL questionnaire, QLQ-C30 Symptom, Functional, and Global Health Items, with regard to the entire sample size and regression predictors at the first (T1) and follow-up (T2) assessment; Table S4: the mean values of the HQoL questionnaire, ELD-14 scales (symptom, function), according to the total sample size and regression predictors at the first (T1) and second (T2) assessment; Table S5: the means and standard deviations (SD) of the evaluated CGA parameters, G8 Screening Tool (G8), Barthel Index (ADL), Instrumental Activity of Daily Living (IADL), and Mini Nutritional Assessment (MNA), according to the regression variables at the first (T1) and second (T2) assessment; Table S6: the patients’ final TNM and UICC classification resulting in palliative treatment; Table S7: the T1 Spearman rank correlations (rs) for the associations between the CGA parameters and questionnaire subscales; and Table S8: the T2 Spearman rank correlations (rs) for the associations between the CGA parameters and questionnaire subscales.

## References

[1] Siegel R.L., Miller K.D., Fuchs H.E., Jemal A. (2022). Cancer statistics, 2022. CA Cancer J. Clin..

[2] Ferlay J., Soerjomataram I., Dikshit R., Eser S., Mathers C., Rebelo M., Parkin D.M., Forman D., Bray F. (2015). Cancer incidence and mortality worldwide: Sources, methods and major patterns in GLOBOCAN 2012. Int. J. Cancer.

[3] Senchak J.J., Fang C.Y., Bauman J.R. (2019). Interventions to improve quality of life (QOL) and/or mood in patients with head and neck cancer (HNC): A review of the evidence. Cancers Head Neck.

[4] Bhatia A., Burtness B. (2023). Treating Head and Neck Cancer in the Age of Immunotherapy: A 2023 Update. Drugs.

[5] Kobayashi K., Hisamatsu K., Suzui N., Hara A., Tomita H., Miyazaki T. (2018). A Review of HPV-Related Head and Neck Cancer. J. Clin. Med..

[6] Johnson D.E., Burtness B., Leemans C.R., Lui V.W.Y., Bauman J.E., Grandis J.R. (2020). Head and neck squamous cell carcinoma. Nat. Rev. Dis. Primers.

[7] Hörmann K., Sadick H. (2013). Role of surgery in the management of head and neck cancer: A contemporary view of the data in the era of organ preservation. J. Laryngol. Otol..

[8] Blanchard P., Baujat B., Holostenco V., Bourredjem A., Baey C., Bourhis J., Pignon J.P. (2011). Meta-analysis of chemotherapy in head and neck cancer (MACH-NC): A comprehensive analysis by tumour site. Radiother. Oncol..

[9] Cohen E.E.W., Soulières D., Le Tourneau C., Dinis J., Licitra L., Ahn M.J., Soria A., Machiels J.P., Mach N., Mehra R. (2019). Pembrolizumab versus methotrexate, docetaxel, or cetuximab for recurrent or metastatic head-and-neck squamous cell carcinoma (KEYNOTE-040): A randomised, open-label, phase 3 study. Lancet.

[10] Weckx A., Riekert M., Grandoch A., Schick V., Zöller J.E., Kreppel M. (2019). Time to recurrence and patient survival in recurrent oral squamous cell carcinoma. Oral Oncol..

[11] Zittel S., Moratin J., Horn D., Metzger K., Ristow O., Engel M., Mrosek J., Freier K., Hoffmann J., Freudlsperger C. (2022). Clinical outcome and prognostic factors in recurrent oral squamous cell carcinoma after primary surgical treatment: A retrospective study. Clin. Oral Investig..

[12] Bonner J.A., Harari P.M., Giralt J., Azarnia N., Shin D.M., Cohen R.B., Jones C.U., Sur R., Raben D., Jassem J. (2006). Radiotherapy plus cetuximab for squamous-cell carcinoma of the head and neck. N. Engl. J. Med..

[13] Burtness B. (2023). First-Line Nivolumab Plus Ipilimumab in Recurrent/Metastatic Head and Neck Cancer-What Happened?. J. Clin. Oncol..

[14] Ferris R.L. (2015). Immunology and Immunotherapy of Head and Neck Cancer. J Clin Oncol.

[15] Ferris R.L., Blumenschein G., Fayette J., Guigay J., Colevas A.D., Licitra L., Harrington K., Kasper S., Vokes E.E., Even C. (2016). Nivolumab for Recurrent Squamous-Cell Carcinoma of the Head and Neck. N. Engl. J. Med..

[16] Luke J.J., Ott P.A. (2015). PD-1 pathway inhibitors: The next generation of immunotherapy for advanced melanoma. Oncotarget.

[17] Burtness B., Harrington K.J., Greil R., Soulières D., Tahara M., de Castro G., Psyrri A., Basté N., Neupane P., Bratland Å. (2019). Pembrolizumab alone or with chemotherapy versus cetuximab with chemotherapy for recurrent or metastatic squamous cell carcinoma of the head and neck (KEYNOTE-048): A randomised, open-label, phase 3 study. Lancet.

[18] Dong L., Wang Y., Yao X., Ren Y., Zhou X. (2023). Novel Insights of Anti-EGFR Therapy in HNSCC: Combined with Immunotherapy or Not?. Curr. Oncol. Rep..

[19] Vermorken J.B., Mesia R., Rivera F., Remenar E., Kawecki A., Rottey S., Erfan J., Zabolotnyy D., Kienzer H.R., Cupissol D. (2008). Platinum-based chemotherapy plus cetuximab in head and neck cancer. N. Engl. J. Med..

[20] Cossu Rocca M., Lorini L., Szturz P., Bossi P., Vermorken J.B. (2023). Recurrent/Metastatic Head and Neck Squamous Cell Carcinoma in Older Patients: Are New Agents Bringing New Hope?. Drugs Aging.

[21] Habbous S., Harland L.T., La Delfa A., Fadhel E., Xu W., Liu F.F., Goldstein D., Waldron J., Huang S.H., O’Sullivan B. (2014). Comorbidity and prognosis in head and neck cancers: Differences by subsite, stage, and human papillomavirus status. Head Neck.

[22] Pilotto A., Cella A., Pilotto A., Daragjati J., Veronese N., Musacchio C., Mello A.M., Logroscino G., Padovani A., Prete C. (2017). Three Decades of Comprehensive Geriatric Assessment: Evidence Coming from Different Healthcare Settings and Specific Clinical Conditions. J. Am. Med. Dir. Assoc..

[23] Bras L., Driessen D., de Vries J., Festen S., van der Laan B., van Leeuwen B.L., de Bock G.H., Halmos G.B. (2020). Patients with head and neck cancer: Are they frailer than patients with other solid malignancies?. Eur. J. Cancer Care.

[24] Veronese N., Custodero C., Demurtas J., Smith L., Barbagallo M., Maggi S., Cella A., Vanacore N., Aprile P.L., Ferrucci L. (2022). Comprehensive geriatric assessment in older people: An umbrella review of health outcomes. Age Ageing.

[25] Haddad R.I., Harrington K., Tahara M., Ferris R.L., Gillison M., Fayette J., Daste A., Koralewski P., Zurawski B., Taberna M. (2023). Nivolumab Plus Ipilimumab Versus EXTREME Regimen as First-Line Treatment for Recurrent/Metastatic Squamous Cell Carcinoma of the Head and Neck: The Final Results of CheckMate 651. J. Clin. Oncol..

[26] Ahern E., Brown T.E., Campbell L., Hughes B.G.M., Banks M., Lin C.Y., Kenny L.M., Bauer J. (2023). Impact of sarcopenia and myosteatosis on survival outcomes for patients with head and neck cancer undergoing curative-intent treatment. Br. J. Nutr..

[27] Kramer B., Wenzel A., Boerger M., Lippert B., Feist K., Petrasch R., Riemann R., Hoermann K., Aderhold C. (2019). Long-Term Quality of Life and Nutritional Status of Patients with Head and Neck Cancer. Nutr. Cancer.

[28] Frick E., Tyroller M., Panzer M. (2007). Anxiety, depression and quality of life of cancer patients undergoing radiation therapy: A cross-sectional study in a community hospital outpatient centre. Eur. J. Cancer Care.

[29] Krebber A.M., Jansen F., Cuijpers P., Leemans C.R., Verdonck-de Leeuw I.M. (2016). Screening for psychological distress in follow-up care to identify head and neck cancer patients with untreated distress. Support Care Cancer.

[30] Winter A., Rasche E., Hartmann S., Schmitter M., Kübler A., Manuel K., Schulz S.M. (2021). Validation of the German-language version of the Liverpool Oral Rehabilitation Questionnaire version 3 and evaluation of oral-health-related quality of life among patients with squamous cell carcinoma of the head and neck. J. Craniomaxillofac. Surg..

[31] Sato N., Koyama S., Mito T., Izumita K., Ishiko R., Yamauchi K., Miyashita H., Ogawa T., Kosaka M., Takahashi T. (2019). Changes in oral health-related quality of life after oral rehabilitation with dental implants in patients following mandibular tumor resection. J. Oral Sci..

[32] Nitschke I., Hahnel S. (2021). Dental care for older people: Opportunities and challenges. Bundesgesundheitsblatt Gesundheitsforschung Gesundheitsschutz.

[33] Nitschke I., Wendland A., Weber S., Jockusch J., Lethaus B., Hahnel S. (2021). Considerations for the Prosthetic Dental Treatment of Geriatric Patients in Germany. J. Clin. Med..

[34] Bellera C.A., Rainfray M., Mathoulin-Pélissier S., Mertens C., Delva F., Fonck M., Soubeyran P.L. (2012). Screening older cancer patients: First evaluation of the G-8 geriatric screening tool. Ann. Oncol..

[35] Kenis C., Decoster L., Van Puyvelde K., De Grève J., Conings G., Milisen K., Flamaing J., Lobelle J.P., Wildiers H. (2014). Performance of two geriatric screening tools in older patients with cancer. J. Clin. Oncol..

[36] Szturz P., Vermorken J.B. (2016). Treatment of Elderly Patients with Squamous Cell Carcinoma of the Head and Neck. Front. Oncol..

[37] Charlson M.E., Pompei P., Ales K.L., MacKenzie C.R. (1987). A new method of classifying prognostic comorbidity in longitudinal studies: Development and validation. J. Chronic Dis..

[38] Glasheen W.P., Cordier T., Gumpina R., Haugh G., Davis J., Renda A. (2019). Charlson Comorbidity Index: ICD-9 Update and ICD-10 Translation. Am. Health Drug Benefits.

[39] Göllnitz I., Inhestern J., Wendt T.G., Buentzel J., Esser D., Böger D., Mueller A.H., Piesold J.U., Schultze-Mosgau S., Eigendorff E. (2016). Role of comorbidity on outcome of head and neck cancer: A population-based study in Thuringia, Germany. Cancer Med..

[40] Singh B., Bhaya M., Stern J., Roland J.T., Zimbler M., Rosenfeld R.M., Har-El G., Lucente F.E. (1997). Validation of the Charlson comorbidity index in patients with head and neck cancer: A multi-institutional study. Laryngoscope.

[41] Oken M.M., Creech R.H., Tormey D.C., Horton J., Davis T.E., McFadden E.T., Carbone P.P. (1982). Toxicity and response criteria of the Eastern Cooperative Oncology Group. Am. J. Clin. Oncol..

[42] Barry E., Galvin R., Keogh C., Horgan F., Fahey T. (2014). Is the Timed Up and Go test a useful predictor of risk of falls in community dwelling older adults: A systematic review and meta-analysis. BMC Geriatr..

[43] Podsiadlo D., Richardson S. (1991). The timed “Up & Go”: A test of basic functional mobility for frail elderly persons. J. Am. Geriatr. Soc..

[44] Goineau A., Campion L., d’Aillières B., Vié B., Ghesquière A., Béra G., Jaffres D., de Laroche G., Magné N., Artignan X. (2018). Comprehensive Geriatric Assessment and quality of life after localized prostate cancer radiotherapy in elderly patients. PLoS ONE.

[45] Han S.H., Cho D., Mohammad R., Jung Y.H., Ahn S.H., Cha W., Jeong W.J. (2022). Use of the comprehensive geriatric assessment for the prediction of postoperative complications in elderly patients with head and neck cancer. Head Neck.

[46] Lawton M.P., Brody E.M. (1969). Assessment of older people: Self-maintaining and instrumental activities of daily living. Gerontologist.

[47] Lübke N., Meinck M., Von Renteln-Kruse W. (2004). The Barthel Index in geriatrics. A context analysis for the Hamburg Classification Manual. Z. Gerontol. Geriatr..

[48] Röhrig G., Becker I., Schulz R.J., Lenzen-Großimlinghaus R., Willschrei P., Gebauer S., Modreker M., Jäger M., Wirth R. (2016). Association between hematologic parameters and functional impairment among geriatric inpatients: Data of a prospective cross-sectional multicenter study (“GeriPrävalenz2013”). Maturitas.

[49] Yamashita K., Yamasaki M., Makino T., Tanaka K., Saito T., Yamamoto K., Takahashi T., Kurokawa Y., Yasunobe Y., Akasaka H. (2023). Preoperative Comprehensive Geriatric Assessment Predicts Postoperative Risk in Older Patients with Esophageal Cancer. Ann. Surg. Oncol..

[50] Salis F., Loddo S., Zanda F., Peralta M.M., Serchisu L., Mandas A. (2022). Comprehensive Geriatric Assessment: Application and correlations in a real-life cross-sectional study. Front. Med..

[51] Vellas B., Guigoz Y., Garry P.J., Nourhashemi F., Bennahum D., Lauque S., Albarede J.L. (1999). The Mini Nutritional Assessment (MNA) and its use in grading the nutritional state of elderly patients. Nutrition.

[52] Folstein M.F., Folstein S.E., McHugh P.R. (1975). “Mini-mental state”. A practical method for grading the cognitive state of patients for the clinician. J. Psychiatr. Res..

[53] Pace-Balzan A., Butterworth C.J., Dawson L.J., Lowe D., Rogers S.N. (2008). The further development and validation of the Liverpool Oral Rehabilitation Questionnaire (LORQ) version 3: A cross-sectional survey of patients referred to a dental hospital for removable prostheses replacement. J. Prosthet. Dent..

[54] Hermann-Lingen C., Buss U., Snaith R.P. (2005). Hospital Anxiety and Depression Scale—Deutsche Version (HADS-D).

[55] Bjelland I., Lie S.A., Dahl A.A., Mykletun A., Stordal E., Kraemer H.C. (2009). A dimensional versus a categorical approach to diagnosis: Anxiety and depression in the HUNT 2 study. Int. J. Methods Psychiatr. Res..

[56] Aaronson N.K., Ahmedzai S., Bergman B., Bullinger M., Cull A., Duez N.J., Filiberti A., Flechtner H., Fleishman S.B., de Haes J.C. (1993). The European Organization for Research and Treatment of Cancer QLQ-C30: A quality-of-life instrument for use in international clinical trials in oncology. J. Natl. Cancer Inst..

[57] Fayers P., Bottomley A. (2002). Quality of life research within the EORTC-the EORTC QLQ-C30. European Organisation for Research and Treatment of Cancer. Eur. J. Cancer.

[58] Fayers P.M., Aaronson N.K., Bjordal K., Groenvold M., Curran D., Bottomley A., on behalf of the EORTC Quality of Life Group (2001). EORTC QLQ-C30 Scoring Manual.

[59] Elaldi R., Roussel L.M., Gal J., Scheller B., Chamorey E., Schiappa R., Lasne-Cardon A., Louis M.Y., Culié D., Dassonville O. (2021). Correlations between long-term quality of life and patient needs and concerns following head and neck cancer treatment and the impact of psychological distress. A multicentric cross-sectional study. Eur. Arch. Otorhinolaryngol..

[60] Kinoshita Y., Izukura R., Kishimoto J., Kanaoka M., Fujita H., Ando K., Nagai S., Akiyoshi S., Tagawa T., Kubo M. (2023). Reliability, validity, and responsiveness of the Japanese version of the EORTC QLQ-ELD14 in evaluating the health-related quality of life of elderly patients with cancer. J. Cancer Res. Clin. Oncol..

[61] Wheelwright S., Darlington A.S., Fitzsimmons D., Fayers P., Arraras J.I., Bonnetain F., Brain E., Bredart A., Chie W.C., Giesinger J. (2013). International validation of the EORTC QLQ-ELD14 questionnaire for assessment of health-related quality of life elderly patients with cancer. Br. J. Cancer.

[62] Gamarra Samaniego M.D.P., Blanquicett C.J., Araujo Castillo R.V., Chavez J.C., Beltrán Garate B.E. (2022). Selected Domains within a Comprehensive Geriatric Assessment in Older Patients with Non-Hodgkin Lymphoma are Highly Associated with Frailty. Clin. Hematol. Int..

[63] Nitschke I., Frank F., Müller-Werdan U., Eckardt-Felmberg R., Stillhart A. (2022). Denture-related problems of patients in acute geriatric care. Z. Gerontol. Geriatr..

[64] Cohen J. (1988). Statistical Power Analysis for the Behavioral Sciences.

[65] Becherini C., Banini M., Desideri I., Salvestrini V., Caprara L., Scotti V., Ganovelli M., Morelli I., Romei A., Livi L. (2023). Clinical outcome of nivolumab in older and frail patients with recurrent/metastatic head and neck squamous cell carcinoma. J. Geriatr. Oncol..

[66] Silver H.J., de Campos Graf Guimaraes C., Pedruzzi P., Badia M., Spuldaro de Carvalho A., Oliveira B.V., Ramos G.H., Dietrich M.S., Pietrobon R. (2010). Predictors of functional decline in locally advanced head and neck cancer patients from south Brazil. Head Neck.

[67] Dewansingh P., Bras L., Ter Beek L., Krijnen W.P., Roodenburg J.L.N., van der Schans C.P., Halmos G.B., Jager-Wittenaar H. (2023). Malnutrition risk and frailty in head and neck cancer patients: Coexistent but distinct conditions. Eur. Arch. Oto-Rhino-Laryngol..

[68] Bøje C.R., Dalton S.O., Primdahl H., Kristensen C.A., Andersen E., Johansen J., Andersen L.J., Overgaard J. (2014). Evaluation of comorbidity in 9388 head and neck cancer patients: A national cohort study from the DAHANCA database. Radiother. Oncol..

[69] Gourin C.G., McAfee W.J., Neyman K.M., Howington J.W., Podolsky R.H., Terris D.J. (2005). Effect of comorbidity on quality of life and treatment selection in patients with squamous cell carcinoma of the head and neck. Laryngoscope.

[70] Hall S.F., Groome P.A., Rothwell D. (2000). The impact of comorbidity on the survival of patients with squamous cell carcinoma of the head and neck. Head Neck.

[71] Leus A.J.G., Haisma M.S., Terra J.B., Sidorenkov G., Festen S., Plaat B.E.C., Halmos G.B., Racz E. (2023). Influence of Frailty and Life Expectancy on Guideline Adherence and Outcomes in Cutaneous Squamous Cell Carcinoma of the Head and Neck: A Prospective Pilot Study. Dermatology.

[72] Piccirillo J.F., Vlahiotis A. (2006). Comorbidity in patients with cancer of the head and neck: Prevalence and impact on treatment and prognosis. Curr. Oncol. Rep..

[73] Harrington K.J., Ferris R.L., Blumenschein G., Colevas A.D., Fayette J., Licitra L., Kasper S., Even C., Vokes E.E., Worden F. (2017). Nivolumab versus standard, single-agent therapy of investigator’s choice in recurrent or metastatic squamous cell carcinoma of the head and neck (CheckMate 141): Health-related quality-of-life results from a randomised, phase 3 trial. Lancet Oncol..

[74] Schmidt H., Nordhausen T., Boese S., Vordermark D., Wheelwright S., Wienke A., Johnson C.D. (2018). Factors Influencing Global Health Related Quality of Life in Elderly Cancer Patients: Results of a Secondary Data Analysis. Geriatrics.

[75] Aghajanzadeh S., Karlsson T., Tuomi L., Engström M., Finizia C. (2023). Trismus, health-related quality of life, and trismus-related symptoms up to 5 years post-radiotherapy for head and neck cancer treated between 2007 and 2012. Support Care Cancer.

[76] Geessink N., Schoon Y., van Goor H., Olde Rikkert M., Melis R. (2017). Frailty and quality of life among older people with and without a cancer diagnosis: Findings from TOPICS-MDS. PLoS ONE.

[77] Rischin D., Harrington K.J., Greil R., Soulières D., Tahara M., de Castro G., Psyrri A., Braña I., Neupane P., Bratland Å. (2022). Pembrolizumab alone or with chemotherapy for recurrent or metastatic head and neck squamous cell carcinoma: Health-related quality-of-life results from KEYNOTE-048. Oral Oncol..

[78] Lavdaniti M., Tilaveridis I., Palitzika D., Kyrgidis A., Triaridis S., Vachtsevanos K., Kosintzi A., Antoniades K. (2022). Quality of Life in Oral Cancer Patients in Greek Clinical Practice: A Cohort Study. J. Clin. Med..

[79] Berg M., Silander E., Bove M., Johansson L., Nyman J., Hammerlid E. (2022). The effect of age on health-related quality of life for head and neck cancer patients up to 1 year after curative treatment. J. Geriatr. Oncol..

[80] Kaufmann A., Schmidt H., Ostheimer C., Ullrich J., Landenberger M., Vordermark D. (2015). Quality of life in very elderly radiotherapy patients: A prospective pilot study using the EORTC QLQ-ELD14 module. Support Care Cancer.

[81] Dholam K., Chouksey G., Dugad J. (2020). Impact of Oral Rehabilitation on Patients with Head and Neck Cancer: Study of 100 Patients with Liverpool Oral Rehabilitation Questionnaire and the Oral Health Impact Profile. Indian J. Otolaryngol. Head Neck Surg..

[82] Li J., Feng K., Ye L., Liu Y., Sun Y., Wu Y. (2022). Influence of radiotherapy on dental implants placed in individuals before diagnosed with head and neck cancer: Focus on implant-bed-specific radiation dosage. Clin. Oral Investig..

[83] Berg M., Silander E., Bove M., Johansson L., Nyman J., Hammerlid E. (2023). Fatigue in Long-Term Head and Neck Cancer Survivors from Diagnosis Until Five Years after Treatment. Laryngoscope.

[84] Goerling U., Gauler T., Dietz A., Grünwald V., Knipping S., Guntinas-Lichius O., Frickhofen N., Lindeman H.W., Fietkau R., Haxel B. (2022). Quality of Life of Patients with Head and Neck Cancer Receiving Cetuximab, Fluorouracil, Cisplatin Comparing to Cetuximab, Fluorouracil, Cisplatin, and Docetaxel within the CEFCID Trial. Oncol. Res. Treat..

[85] Hinz A., Schwarz R. (2001). Anxiety and depression in the general population: Normal values in the Hospital Anxiety and Depression Scale. Psychother. Psychosom. Med. Psychol..

[86] Singh P., Bennett B., Bailey T., Taylor-Stokes G., Rajkovic I., Contente M., Curtis S., Curtis C. (2021). Real-world study of the impact of recurrent/metastatic squamous cell carcinoma of the head and neck (R/M SCCHN) on quality of life and productivity in Europe. BMC Cancer.

[87] Nitschke I., Majdani M., Sobotta B.A., Reiber T., Hopfenmüller W. (2010). Dental care of frail older people and those caring for them. J. Clin. Nurs..

[88] Dholam K.P., Dugad J.A., Sadashiva K.M. (2017). Impact of oral rehabilitation on patients with head and neck cancer: A study using the Liverpool Oral Rehabilitation Questionnaire and the Oral Health Impact Profile-14. J. Prosthet. Dent..

[89] Hammermüller C., Hinz A., Dietz A., Wichmann G., Pirlich M., Berger T., Zimmermann K., Neumuth T., Mehnert-Theuerkauf A., Wiegand S. (2021). Depression, anxiety, fatigue, and quality of life in a large sample of patients suffering from head and neck cancer in comparison with the general population. BMC Cancer.

[90] Mountzios G. (2015). Optimal management of the elderly patient with head and neck cancer: Issues regarding surgery, irradiation and chemotherapy. World J. Clin. Oncol..

[91] Deschodt M., Flamaing J., Haentjens P., Boonen S., Milisen K. (2013). Impact of geriatric consultation teams on clinical outcome in acute hospitals: A systematic review and meta-analysis. BMC Med..

[92] Vigneswaran N., Williams M.D. (2014). Epidemiologic trends in head and neck cancer and aids in diagnosis. Oral Maxillofac. Surg. Clin. N. Am..

[93] Parker S.G., McCue P., Phelps K., McCleod A., Arora S., Nockels K., Kennedy S., Roberts H., Conroy S. (2018). What is Comprehensive Geriatric Assessment (CGA)? An umbrella review. Age Ageing.

