# Supplementary material for: Comprehensive Geriatric Assessment and Quality of Life Aspects in Patients with Recurrent/Metastatic Head and Neck Squamous Cell Carcinoma (HNSCC)
Source: J Clin Med. 2023 Sep 3;12(17):5738. doi: 10.3390/jcm12175738 (PMC10488489; doi:10.3390/jcm12175738)
Supplement: Supplementary file 1 [file jcm-12-05738-s001.zip › Table S1.pdf]

Table S1. Mean values of the OHRQoL measured by LORQv3 (Liverpool Oral Rehabilitation Questionnaire version 3) with regard to the entire patient population and regression predictors at baseline (T1) and follow-up (T2) assessment. SD: Standard deviation.

**Time of assessment**			**T1**						**T2**					
**Variables**	**Section/Domain**		Total	Section 1	Oral Function	Orofacial Appearance	Social Interaction	Section 2	Total	Section 1	Oral Function	Orofacial Appearance	Social Interaction	Section 2
Total		Mean	2.28	2.51	2.52	2.49	0.16	3.13	2.62	2.69	2.73	2.58	0.18	2.88
		SD	0.63	0.67	0.65	1.03	0.08	1.45	0.82	0.77	0.77	1.25	0.07	1.38
Age	< 65 years	Mean	2.40	2.55	2.42	2.96	0.16	2.88	3.12	3.12	2.97	3.55	0.21	2.88
		SD	0.75	0.88	0.79	1.27	0.09	1.15	0.74	0.83	0.81	1.01	0.07	1.15
	≥ 65 years	Mean	2.14	2.48	2.58	2.23	0.16	3.24	2.40	2.50	2.62	2.14	0.17	3.24
		SD	0.54	0.56	0.58	0.82	0.07	1.62	0.75	0.69	0.77	1.12	0.07	1.62
Need for prosthetic treatment	Yes	Mean	2.34	2.55	2.55	2.57	0.16	3.58	2.79	2.80	2.83	2.65	0.19	3.58
		SD	0.66	0.71	0.69	1.10	0.08	1.28	0.81	0.81	0.79	1.35	0.07	1.28
	No	Mean	1.94	2.25	2.33	2.00	0.16	1.61	1.92	2.25	2.28	2.25	0.13	1.61
		SD	0.23	0.24	0.38	0.25	0.04	0.92	0.31	0.30	0.57	0.75	0.00	0.92
Primary HNSCC therapy	surgery and radiotherapy±chemotherapy	Mean	2.34	2.56	2.58	2.55	0.16	3.37	2.77	2.84	2.90	2.63	0.19	3.23
		SD	0.69	0.73	0.70	1.11	0.08	1.44	0.82	0.77	0.76	1.31	0.07	1.19
	surgery only	Mean	2.03	2.29	2.29	2.25	0.17	2.33	1.96	2.08	2.00	2.33	0.13	2.00
		SD	0.09	0.31	0.31	0.68	0.04	1.44	2.26	0.43	0.22	1.15	0.00	1.89
Oral functional capacity	RCL1	Mean	-	-	-	-	-	-	1.76	2.04	2.19	1.67	0.11	2.72
		SD	-	-	-	-	-	-	0.25	0.45	0.63	0.52	0.04	1.18
	RCL2	Mean	1.87	2.01	2.12	1.75	0.12	2.72	1.83	1.94	2.08	1.50	0.13	
		SD	0.25	0.29	0.33	0.40	0.06	1.18	0.24	0.25	0.47	0.35	0.00	
	RCL3	Mean	2.32	2.59	2.58	2.64	0.17	3.25	2.90	2.93	2.92	2.93	0.21	3.26
		SD	0.56	0.62	0.63	1.05	0.08	1.56	0.71	0.68	0.73	1.28	0.07	1.65
	RCL4	Mean	3.82	3.82	3.75	4.00	0.27		3.82	3.82	3.75	4.00	0.27	-
		SD	-	-	-	-	-	-	-	-	-	-	-	-
