# Supplementary material for: Comprehensive Geriatric Assessment and Quality of Life Aspects in Patients with Recurrent/Metastatic Head and Neck Squamous Cell Carcinoma (HNSCC)
Source: J Clin Med. 2023 Sep 3;12(17):5738. doi: 10.3390/jcm12175738 (PMC10488489; doi:10.3390/jcm12175738)
Supplement: Supplementary file 1 [file jcm-12-05738-s001.zip › Table S2.pdf]

Table S2. Mean values of psychological questionnaire HADS with regard to the entire patient population and regression predictors at baseline (T1) and follow-up (T2) assessment. SD: Standard deviation.

	**Time of assessment**		**T1**		**T2**	
			**Anxiety**	**Depression**	**Anxiety**	**Depression**
Total		Mean	6.67	8.57	6.18	8.35
		SD	3.84	3.74	4.08	3.86
Age	< 65 years	Mean	6.63	8.88	8.00	9.00
		SD	3.25	3.68	3.58	4.90
	≥ 65 years	Mean	6.69	8.38	5.18	8.00
		SD	4.29	3.91	4.14	3.38
Need for prosthetic treatment	Yes	Mean	6.89	8.78	6.43	8.64
		SD	3.98	3.95	4.26	3.82
	No	Mean	5.33	7.33	5.00	7.00
		SD	3.06	2.08	3.61	4.58
Primary HNSCC therapy	surgery and radio-therapy± chemo-therapy	Mean	6.76	8.88	6.79	8.21
		SD	3.98	4.04	3.95	4.19
	surgery only	Mean	6.25	7.25	3.33	9.00
		SD	3.69	1.71	4.16	2.00
Oral functional capacity	RCL1	Mean	-		3.67	5.00
		SD	-	-	2.52	3.00
	RCL2	Mean	5.40	6.60	3.50	5.50
		SD	3.97	3.51	3.54	2.12
	RCL3	Mean	6.73	8.87	6.73	9.36
		SD	3.73	3.58	3.98	3.64
	RCL4	Mean	12.00	14.00	13.00	13.00
		SD	-	-	-	-
