# Supplementary material for: Comprehensive Geriatric Assessment and Quality of Life Aspects in Patients with Recurrent/Metastatic Head and Neck Squamous Cell Carcinoma (HNSCC)
Source: J Clin Med. 2023 Sep 3;12(17):5738. doi: 10.3390/jcm12175738 (PMC10488489; doi:10.3390/jcm12175738)
Supplement: Supplementary file 1 [file jcm-12-05738-s001.zip › Table S3.pdf]

Table S3. Mean values of the HQoL questionnaire QLQ-C30 Symptom, Functional, and Global Health Items with regard to the entire sample size and regression predictors at first (T1) and follow-up (T2) assessment. SD: Standard deviation.

Variable	Scale		Physical Functioning		Role Functioning	
	Assessment time		T1	T2	T1	T2
Total		Mean	62.67	57.50	43.33	37.50
		SD	21.99	25.17	33.07	30.12
Age	< 65 years	Mean	62.86	64.00	38.10	33.33
		SD	20.68	26.92	31.50	40.82
	≥ 65 years	Mean	62.56	54.55	46.15	39.39
		SD	23.50	25.09	34.80	26.11
Need for prosthetic treatment	Yes	Mean	59.22	55.90	41.18	37.18
		SD	21.97	27.15	34.42	32.03
	No	Mean	82.22	64.44	55.56	38.89
		SD	7.70	15.40	25.46	25.46
Primary HNSCC therapy	surgery and radio-therapy± chemo-therapy	Mean	64.17	62.05	42.71	43.59
		SD	21.06	21.84	28.52	29.30
	surgery only	Mean	56.67	37.78	45.83	11.11
		SD	28.02	34.21	53.36	19.25
Oral functional capacity	RCL1	Mean	-	66.67	-	50.00
		SD	-	11.55	-	28.87
	RCL2	Mean	76.00	68.00	53.33	50.00
		SD	5.96	8.69	21.73	23.57
	RCL3	Mean	58.10	52.67	39.29	35.00
		SD	24.59	30.54	37.33	31.87
	RCL4	Mean	60.00	53.33	50.00	0.00
		SD	-	-	-	-

Variable	Scale		Emotional Functioning		Cognitive Functioning	
	Assessment time		T1	T2	T1	T2
Total		Mean	55.00	59.38	70.00	69.79
		SD	21.19	28.36	23.32	23.74
Age	< 65 years	Mean	48.81	51.67	66.67	76.67
		SD	23.29	25.95	33.33	27.89
	≥ 65 years	Mean	58.33	62.88	71.79	66.67
		SD	20.13	29.90	17.19	22.36
Need for prosthetic treatment	Yes	Mean	52.94	55.13	70.59	69.23
		SD	22.23	29.57	24.67	26.22
	No	Mean	66.67	77.78	66.67	72.22
		SD	8.33	12.73	16.67	9.62
Primary HNSCC therapy	surgery and radio-therapy± chemo-therapy	Mean	54.17	56.41	71.88	73.08
		SD	23.17	28.50	25.62	21.01
	surgery only	Mean	58.33	72.22	62.50	55.56
		SD	11.79	29.27	8.33	34.69
Oral functional capacity	RCL1	Mean	-	72.22	-	72.22
		SD	-	17.35	-	9.62
	RCL2	Mean	70.00	78.33	70.00	76.67
		SD	17.28	17.28	21.73	9.13
	RCL3	Mean	50.60	54.17	67.86	66.67
		SD	21.05	27.85	23.99	29.40
	RCL4	Mean	41.67	16.67	100.00	66.67
		SD	-	-	-	-

Variable	Scale		Social Functioning		Dyspnoe	
	Assessment time		T1	T2	T1	T2
Total		Mean	45.83	39.58	28.33	43.75
		SD	37.02	35.42	27.09	26.44
Age	< 65 years	Mean	45.24	46.67	28.57	33.33
		SD	39.34	44.72	29.99	33.33
	≥ 65 years	Mean	46.15	36.36	28.21	48.48
		SD	37.36	32.33	26.69	22.92
Need for prosthetic treatment	Yes	Mean	42.16	38.46	27.45	43.59
		SD	35.90	39.31	26.97	28.50
	No	Mean	66.67	44.44	33.33	44.44
		SD	44.10	9.62	33.33	19.25
Primary HNSCC therapy	surgery and radio-therapy± chemo-therapy	Mean	46.88	46.15	31.25	48.72
		SD	37.13	35.46	25.73	25.88
	surgery only	Mean	41.67	11.11	16.67	22.22
		SD	41.94	19.25	33.33	19.25
Oral functional capacity	RCL1	Mean	-	61.11	-	55.56
		SD	-	19.25	-	19.25
	RCL2	Mean	63.33	66.67	40.00	53.33
		SD	32.06	16.67	14.91	18.26
	RCL3	Mean	42.86	30.00	21.43	36.67
		SD	37.39	35.83	28.06	29.19
	RCL4	Mean	0.00	0.00	66.67	66.67
		SD	-	-	-	-

Variable	Scale		Insomnia		Appetite loss	
	Assessment time		T1	T2	T1	T2
Total		Mean	41.67	50.00	30.00	37.50
		SD	26.21	32.20	37.31	31.91
Age	< 65 years	Mean	47.62	60.00	33.33	40.00
		SD	32.53	36.51	38.49	27.89
	≥ 65 years	Mean	38.46	45.45	28.21	36.36
		SD	22.96	30.81	38.12	34.82
Need for prosthetic treatment	Yes	Mean	37.25	53.85	29.41	35.90
		SD	26.04	32.03	35.12	31.80
	No	Mean	66.67	33.33	33.33	44.44
		SD	0.00	33.33	57.74	38.49
Primary HNSCC therapy	surgery and radio-therapy± chemo-therapy	Mean	45.83	51.28	25.00	33.33
		SD	23.96	32.25	31.03	27.22
	surgery only	Mean	25.00	44.44	50.00	55.56
		SD	31.91	38.49	57.74	50.92
Oral functional capacity	RCL1	Mean	-	33.33	-	33.33
		SD	-	33.33	-	33.33
	RCL2	Mean	40.00	26.67	13.33	26.67
		SD	27.89	27.89	18.26	27.89
	RCL3	Mean	40.48	56.67	38.10	46.67
		SD	26.73	27.44	41.05	32.20
	RCL4	Mean	66.67	100.00	0.00	0.00
		SD	-	-	-	-

Variable	Scale		Nausea		Constipation	
	Assessment time		T1	T2	T1	T2
Total		Mean	4.17	4.17	25.00	22.92
		SD	7.40	7.45	30.35	33.82
Age	< 65 years	Mean	2.38	3.33	23.81	13.33
		SD	6.30	7.45	25.20	29.81
	≥ 65 years	Mean	5.13	4.55	25.64	27.27
		SD	8.01	7.78	33.76	35.96
Need for prosthetic treatment	Yes	Mean	4.90	5.13	27.45	25.64
		SD	7.83	8.01	31.70	36.40
	No	Mean	0.00	0.00	11.11	11.11
		SD	0.00	0.00	19.25	19.25
Primary HNSCC therapy	surgery and radio-therapy± chemo-therapy	Mean	4.17	5.13	25.00	25.64
		SD	7.45	8.01	31.03	36.40
	surgery only	Mean	4.17	0.00	25.00	11.11
		SD	8.33	0.00	31.91	19.25
Oral functional capacity	RCL1	Mean	-	0.00	-	22.22
		SD	-	0.00	-	38.49
	RCL2	Mean	0.00	0.00	6.67	20.00
		SD	0.00	0.00	14.91	29.81
	RCL3	Mean	5.95	6.67	33.33	26.67
		SD	8.29	8.61	32.03	37.84
	RCL4	Mean	0.00	0.00	0.00	0.00
		SD	-	-	-	-

Variable	Scale		Diarrhoe		Fatigue	
	Assessment time		T1	T2	T1	T2
Total		Mean	5.00	22.92	46.67	61.11
		SD	12.21	33.82	20.90	20.29
Age	< 65 years	Mean	4.76	0.00	49.21	53.33
		SD	12.60	0.00	21.14	18.26
	≥ 65 years	Mean	5.13	33.33	45.30	64.65
		SD	12.52	36.51	21.50	20.98
Need for prosthetic treatment	Yes	Mean	5.88	25.64	46.41	61.54
		SD	13.10	36.40	20.50	20.60
	No	Mean	0.00	11.11	48.15	59.26
		SD	0.00	19.25	27.96	23.13
Primary HNSCC therapy	surgery and radio-therapy± chemo-therapy	Mean	6.25	20.51	51.39	56.41
		SD	13.44	28.99	18.98	18.40
	surgery only	Mean	0.00	33.33	27.78	21.48
		SD	0.00	57.74	19.25	16.97
Oral functional capacity	RCL1	Mean	-	33.33	-	51.85
		SD	-	33.33	-	23.13
	RCL2	Mean	6.67	26.67	48.89	46.67
		SD	14.91	27.89	18.59	18.26
	RCL3	Mean	4.76	23.33	44.44	67.78
		SD	12.10	38.65	22.22	19.21
	RCL4	Mean	0.00	0.00	66.67	66.67
		SD	-	-	-	-

Variable	Scale		Pain		Financial Difficulties	
	Assessment time		T1	T2	T1	T2
Total		Mean	47.50	43.75	38.33	33.33
		SD	29.75	34.36	37.89	38.49
Age	< 65 years	Mean	45.24	40.00	57.14	40.00
		SD	32.93	43.46	41.79	43.46
	≥ 65 years	Mean	48.72	45.45	28.21	30.30
		SD	29.24	31.70	32.90	37.87
Need for prosthetic treatment	Yes	Mean	50.00	44.87	43.14	41.03
		SD	31.18	35.61	38.67	38.86
	No	Mean	33.33	38.89	11.11	0.00
		SD	16.67	34.69	19.25	0.00
Primary HNSCC therapy	surgery and radio-therapy± chemo-therapy	Mean	51.04	41.03	41.67	33.33
		SD	28.20	31.63	39.44	36.00
	surgery only	Mean	33.33	55.56	25.00	33.33
		SD	36.00	50.92	31.91	57.74
Oral functional capacity	RCL1	Mean	-	33.33	-	11.11
		SD	-	28.87	-	19.25
	RCL2	Mean	40.00	30.00	13.33	20.00
		SD	19.00	21.73	18.26	29.81
	RCL3	Mean	48.81	48.33	47.62	43.33
		SD	33.63	39.64	40.75	41.72
	RCL4	Mean	66.67	66.67	33.33	0.00
		SD	-	-	-	-

Variable	Scale		Global Health	
	Assessment time		T1	T2
Total		Mean	48.33	46.87
		SD	20.34	24.32
Age	< 65 years	Mean	53.57	51.67
		SD	20.33	20.75
	≥ 65 years	Mean	45.51	44.70
		SD	20.59	26.42
Need for prosthetic treatment	Yes	Mean	44.61	46.15
		SD	18.39	26.27
	No	Mean	69.44	50.00
		SD	20.97	16.67
Primary HNSCC therapy	surgery and radio-therapy± chemo-therapy	Mean	47.92	51.92
		SD	18.13	22.09
	surgery only	Mean	50.00	25.00
		SD	31.18	25.00
Oral functional capacity	RCL1	Mean	-	58.33
		SD	-	22.05
	RCL2	Mean	58.33	58.33
		SD	8.33	0.00
	RCL3	Mean	45.83	42.50
		SD	22.82	27.62
	RCL4	Mean	33.33	33.33
		SD	-	-
