# Supplementary material for: Comprehensive Geriatric Assessment and Quality of Life Aspects in Patients with Recurrent/Metastatic Head and Neck Squamous Cell Carcinoma (HNSCC)
Source: J Clin Med. 2023 Sep 3;12(17):5738. doi: 10.3390/jcm12175738 (PMC10488489; doi:10.3390/jcm12175738)
Supplement: Supplementary file 1 [file jcm-12-05738-s001.zip › Table S4.pdf]

Table S4. Mean values of the HQoL questionnaire ELD-14 scales (symptom, function) according to the total sample size and regression predictors at first (T1) and second (T2) assessment. SD: Standard deviation.

Variable	Scale		Maintaining Purpose		Family Support	
	Assessment time		T1	T2	T1	T2
Total		Mean	63.33	51.96	55.00	50.98
		SD	28.41	27.56	39.40	35.59
Age	< 65 years	Mean	57.14	50.00	52.38	50.00
		SD	21.21	25.82	42.41	27.89
	≥ 65 years	Mean	66.67	53.03	56.41	51.52
		SD	31.91	29.64	39.40	40.45
Need for prosthetic treatment	Yes	Mean	60.78	52.38	54.90	57.14
		SD	28.83	28.39	38.98	33.15
	No	Mean	77.78	50.00	55.56	22.22
		SD	25.46	28.87	50.92	38.49
Primary HNSCC therapy	surgery and radio-therapy± chemo-therapy	Mean	61.46	50.00	45.83	42.86
		SD	30.26	26.95	38.25	33.15
	surgery only	Mean	70.83	61.11	91.67	88.89
		SD	20.97	34.69	16.67	19.25
Oral functional capacity	RCL1	Mean	-	61.11	-	11.11
		SD	-	25.46	-	19.25
	RCL2	Mean	66.67	66.67	40.00	33.33
		SD	42.49	26.35	43.46	40.82
	RCL3	Mean	63.10	45.45	59.52	60.61
		SD	24.62	27.98	39.61	32.72
	RCL4	Mean	50.00	50.00	66.67	33.33
		SD	-	-	-	-

Variable	Scale		Joint Stiffness		Mobility	
	Assessment time		T1	T2	T1	T2
Total		Mean	46.67	47.06	50.00	47.71
		SD	29.42	31.31	30.48	22.49
Age	< 65 years	Mean	38.10	38.89	46.03	42.59
		SD	35.63	25.09	30.38	19.14
	≥ 65 years	Mean	51.28	51.52	52.14	50.51
		SD	25.88	34.52	31.55	24.53
Need for prosthetic treatment	Yes	Mean	47.06	50.00	54.25	50.79
		SD	29.01	31.35	30.65	23.35
	No	Mean	44.44	33.33	25.93	33.33
		SD	38.49	33.33	16.97	11.11
Primary HNSCC therapy	surgery and radio-therapy± chemo-therapy	Mean	45.83	47.62	48.61	45.24
		SD	31.91	28.39	29.78	19.72
	surgery only	Mean	50.00	44.44	55.56	59.26
		SD	19.25	50.92	37.41	35.72
Oral functional capacity	RCL1	Mean	-	55.56	-	29.63
		SD	-	50.92	-	6.42
	RCL2	Mean	46.67	46.67	37.78	31.11
		SD	38.01	38.01	35.66	4.97
	RCL3	Mean	45.24	45.45	53.97	55.56
		SD	28.06	30.81	29.83	24.34
	RCL4	Mean	66.67	66.67	55.56	44.44
		SD	-	-	-	-

Variable	Scale		Worries about others		Future worries	
	Assessment time		T1	T2	T1	T2
Total		Mean	59.17	49.02	62.22	56.21
		SD	29.36	37.95	27.78	25.30
Age	< 65 years	Mean	54.76	52.78	60.32	57.41
		SD	15.85	38.61	29.99	24.76
	≥ 65 years	Mean	61.54	46.97	63.25	55.56
		SD	34.95	39.31	27.74	26.76
Need for prosthetic treatment	Yes	Mean	63.73	54.76	66.01	59.52
		SD	25.16	38.36	27.35	26.73
	No	Mean	33.33	22.22	40.74	40.74
		SD	44.10	25.46	23.13	15493
Primary HNSCC therapy	surgery and radio-therapy± chemo-therapy	Mean	58.33	52.38	62.50	54.76
		SD	26.53	34.50	27.78	24.45
	surgery only	Mean	62.50	33.33	61.11	62.96
		SD	43.83	57.74	32.08	33.95
Oral functional capacity	RCL1	Mean	-	44.44	-	37.04
		SD	-	25.46	-	12.83
	RCL2	Mean	46.67	43.33	46.67	42.22
		SD	29.81	19.00	19.88	16.48
	RCL3	Mean	63.10	50.00	65.87	58.59
		SD	30.08	45.95	45106	24.89
	RCL4	Mean	66.67	66.67	88.89	100.00
		SD	-	-	-	-

Variable	Scale		Burden of Illness	
	Assessment time		T1	T2
Total		Mean	65.00	62.75
		SD	18.65	23.22
Age	< 65 years	Mean	61.90	61.11
		SD	23.00	27.22
	≥ 65 years	Mean	66.67	63.64
		SD	16.67	22.13
Need for prosthetic treatment	Yes	Mean	65.69	64.29
		SD	19.96	25.20
	No	Mean	61.11	55.56
		SD	9.62	9.61
Primary HNSCC therapy	surgery and radio-therapy± chemo-therapy	Mean	64.58	60.71
		SD	20.07	23.21
	surgery only	Mean	66.67	72.22
		SD	13.61	25.46
Oral functional capacity	RCL1	Mean	-	50.00
		SD	-	0.00
	RCL2	Mean	56.67	43.33
		SD	19.00	41518
	RCL3	Mean	65.48	69.70
		SD	16.62	23.35
	RCL4	Mean	100.00	83.33
		SD	-	-
