# Supplementary material for: Comprehensive Geriatric Assessment and Quality of Life Aspects in Patients with Recurrent/Metastatic Head and Neck Squamous Cell Carcinoma (HNSCC)
Source: J Clin Med. 2023 Sep 3;12(17):5738. doi: 10.3390/jcm12175738 (PMC10488489; doi:10.3390/jcm12175738)
Supplement: Supplementary file 1 [file jcm-12-05738-s001.zip › Table S5.pdf]

Table S5. Means and standard deviations (SD) of evaluated CGA parameters G8 Screening Tool (G8), Barthel Index (ADL), Instrumental Activity of Daily Living (IADL), and Mini Nutritional Assessment (MNA) according to the regression variables at first (T1) and second (T2) assessment.

Time of assessment			T1					T2				
**Variables**		**Parameter**	**G8**	**ADL**	**IADL**	**MMST**	**MNA**	**G8**	**ADL**	**IADL**	**MMST**	**MNA**
Total		Mean	9.64	91.19	5.86	25.38	17.17	9.28	53.30	4.35	22.20	16.58
		SD	2.53	13.41	2.15	4.03	2.91	3.12	17.34	3.31	9.99	5.03
Age	< 65 years	Mean	10.03	90.28	5.61	25.39	17.06	9.09	72.35	3.82	21.76	16.03
		SD	2.42	14.19	2.20	3.78	3.03	3.09	36.66	3.30	10.49	5.25
	≥ 65 years	Mean	7.17	96.67	7.33	25.33	17.83	10.33	95.00	7.33	24.67	19.67
		SD	1.04	5.77	1.15	6.43	2.47	3.79	5.00	1.15	7.57	1.61
Need for prosthetic treatment	Yes	Mean	10.19	96.88	6.38	25.63	17.88	8.94	73.13	4.13	20.00	15.31
		SD	2.17	8.84	1.77	4.03	2.03	3.36	42.42	3.60	11.83	5.82
	No	Mean	9.27	87.69	5.54	25.23	16.73	9.50	77.50	4.50	23.67	17.42
		SD	2.67	14.81	2.37	4.19	3.35	3.08	30.41	3.26	8.79	4.49
Primary HNSCC therapy	surgery and radio-therapy± chemo-therapy	Mean	9.97	93.82	6.18	26.59	17.41	9.65	77.35	4.59	22.76	16.88
		SD	2.29	10.08	2.04	3.36	2.48	3.22	36.28	3.39	10.70	5.23
	surgery only	Mean	8.13	80.00	4.50	20.25	16.13	7.17	66.67	3.00	19.00	14.83
		SD	3.07	21.21	2.38	2.22	4.70	1.26	27.54	3.00	3.61	4.01
Oral functional capacity	RCL1	Mean	-	-	-	-	-	13.00	95.00	8.00	28.67	21.33
		SD	-	-	-	-	-	1.00	5.00	0.00	1.15	2.75
	RCL2	Mean	9.90	97.00	7.80	28.40	17.50	12.00	100.00	8.00	28.50	22.50
		SD	2.92	4.47	0.45	1.67	3.30	0.00	0.00	0.00	2.12	1.41
	RCL3	Mean	9.43	88.67	5.07	24.07	17.07	8.11	66.43	2.79	19.36	14.71
		SD	2.48	15.06	2.05	3.97	2.99	2.88	37.95	2.69	10.77	4.61
	RCL4	Mean	11.00	100.00	8.00	30.00	17.00	9.00	100.00	8.00	30.00	16.50
		SD	-	-	-	-	-	-	-	-	-	-
