# Supplementary material for: Comprehensive Geriatric Assessment and Quality of Life Aspects in Patients with Recurrent/Metastatic Head and Neck Squamous Cell Carcinoma (HNSCC)
Source: J Clin Med. 2023 Sep 3;12(17):5738. doi: 10.3390/jcm12175738 (PMC10488489; doi:10.3390/jcm12175738)
Supplement: Supplementary file 1 [file jcm-12-05738-s001.zip › Table S6.pdf]

**Table S6.** Patients final TNM and UICC classification resulting in palliative treatment.

| Patient No. | TNM resulting in Immunotherapy | UICC |
|---|---|---|
| **1** | pT2 NX L0 V0 Pn0 G2 R1 | II |
| **2** | pT3 N0 M0 G1-2 L0 V0 R0 | III |
| **3** | cT3 cN2c pM1 R2 | IV |
| **4** | rpT3 N3b M1 G3 L1 V0 R0 | IV |
| **5** | rpT2 N3b L0 V0 Pn0 G2 R0 | IV |
| **6** | pT4a NX L0 V0 Pn1 G2-3 RX | IV |
| **7** | rpT2 N0 L0 V0 Pn1 G3 R0 | II |
| **8** | pT1 NX, L0, V0, Pn0, G2, R0 | I |
| **9** | pT4a NX M0 G2 L0 V0 Pn1 R2 | IV |
| **10** | rpT4a NX L0 V0 Pn1 G3 RX | IV |
| **11** | cT4 N3b M0 G2 L1 V1 RX | IV |
| **12** | cT4 N2c MX G3 RX | IV |
| **13** | cT4a N0 M0 L0 V0 Pn0 G2-3 RX | IV |
| **14** | cT4 N0 M0 G1 L0 V0 Pn0 RX | IV |
| **15** | cT3 N0 M0 RX G3 L0 V0 Pn0 RX | III |
| **16** | rpT3 N2 L1 V1 Pn1 G3 R1 | IV |
| **17** | pT4a NX M0 G2-3 L0 V0 R0 | IV |
| **18** | pT4a N1 M0 G3 L1 V0 R1 | IV |
| **19** | rpT3 N2b cM1 L1 V0 Pn1 G2 R0 | IV |
| **20** | cT4 N2b cM0 G2 L0 V0 Pn1 RX | IV |
| **21** | pT2 NX L0 V0 Pn0 G2 R1 | II |
