# Supplementary material for: Comprehensive Geriatric Assessment and Quality of Life Aspects in Patients with Recurrent/Metastatic Head and Neck Squamous Cell Carcinoma (HNSCC)
Source: J Clin Med. 2023 Sep 3;12(17):5738. doi: 10.3390/jcm12175738 (PMC10488489; doi:10.3390/jcm12175738)
Supplement: Supplementary file 1 [file jcm-12-05738-s001.zip › Table S7.pdf]

Table S7. T1 Spearman rank correlations (r_s) for associations between CGA parameters and questionnaire subscales. Significant p-values ($\alpha \leq .05$) are marked bold.

**CGA Parameter**	**CCI**		**G8**		**ECOG**		**TUG**	
**Questionnaire**	r_s	p	r_s	p	r_s	p	r_s	p
HADS-A	-0.079	0.734	-0.034	0.882	0.028	0.906	-0.428	0.053
HADS-D	-0.205	0.373	0.155	0.503	0.036	0.876	-0.353	0.117
LORQv3 total	0.307	0.188	0.108	0.651	0.069	0.772	-0.127	0.592
LORQv3 section1	-0.023	0.922	0.111	0.640	0.031	0.895	-0.237	0.315
LORQv3 Oral Function	-0.002	0.994	0.129	0.587	-0.052	0.829	-0.200	0.397
LORQv3 Orofacial Appearance	0.066	0.783	-0.141	0.552	0.249	0.291	-0.276	0.240
LORQv3 Social Interaction	0.057	0.810	0.143	0.549	0.107	0.652	-0.037	0.876
LORQv3 Section2	0.324	0.280	0.419	0.154	-0.006	0.984	0.134	0.663
EORTC-ELD-14 JointStiffness	0.354	0.125	0.074	0.757	-0.009	0.970	-0.067	0.778
EORTC-ELD-14 Mobility	0.337	0.146	0.426	0.061	0.326	0.161	0.185	0.436
EORTC-ELD-14 FamilySupport	0.239	0.311	0.020	0.932	0.020	0.932	-0.113	0.635
EORTC-ELD-14 Worries about others	0.513	**0.021**	0.226	0.339	0.392	0.087	0.074	0.756
EORTC-ELD-14 Future worries	0.346	0.135	0.019	0.938	0.213	0.368	-0.193	0.415
EORTC-ELD-14 Maintaining Purpose	0.014	0.952	-0.387	0.092	0.182	0.442	0.000	1.000
EORTC-ELD-14 Burden of illness	0.397	0.083	0.218	0.356	0.043	0.856	-0.132	0.580
EORTC-C30 Physical Functioning	-0.236	0.317	-0.334	0.150	-0.454	**0.044**	-0.128	0.590
EORTC-C30 Role Functioning	-0.263	0.262	-0.328	0.157	-0.313	0.179	0.185	0.435
EORTC-C30 Emotional Functioning	-0.279	0.233	-0.189	0.424	-0.340	0.142	0.366	0.112
EORTC-C30 Cognitive Functioning	0.189	0.426	0.176	0.459	-0.131	0.582	0.093	0.696
EORTC-C30 Social Functioning	-0.451	**0.046**	0.107	0.653	-0.526	0.017	0.101	0.670
EORTC-C30 Dyspnoe	0.246	0.296	0.101	0.671	-0.170	0.474	-0.262	0.265

EORTC-C30 Insomnia	0.119	0.618	-0.223	0.346	-0.048	0.840	-0.069	0.772
EORTC-C30 Appetite loss	-0.231	0.327	0.042	0.861	0.394	0.086	0.245	0.297
EORTC-C30 Nausea	0.372	0.106	0.221	0.349	0.401	0.080	0.182	0.444
EORTC-C30 Constipation	0.194	0.412	-0.016	0.948	0.266	0.256	0.000	1.000
EORTC-C30 Diarrhoe	-0.075	0.753	0.085	0.721	0.338	0.145	0.308	0.186
EORTC-C30 Fatigue	0.090	0.707	0.050	0.833	0.070	0.769	-0.092	0.699
EORTC-C30 Pain	0.377	0.101	0.161	0.498	0.145	0.542	-0.047	0.845
EORTC-C30 Financial difficulties	0.248	0.292	0.031	0.896	0.563	0.010	-0.086	0.719
EORTC-C30 QoL/Global health	-0.362	0.117	-0.276	0.238	-0.449	**0.047**	-0.102	0.670

CGA Parameter	ADL		IADL		MMSE		MNA	
**Questionnaire**	r_s	p	r_s	p	r_s	p	r_s	p
HADS-A	0.084	0.718	0.080	0.731	-0.097	0.677	-0.146	0.527
HADS-D	0.053	0.820	-0.071	0.761	-0.072	0.757	0.062	0.789
LORQv3 total	-0.062	0.795	-0.208	0.379	-0.035	0.883	0.213	0.366
LORQv3 section1	0.203	0.390	-0.081	0.734	0.095	0.691	0.000	0.999
LORQv3 Oral Function	0.180	0.448	-0.094	0.695	0.151	0.525	0.058	0.808
LORQv3 Orofacial Appearance	0.092	0.700	0.044	0.855	-0.186	0.431	-0.335	0.148
LORQv3 Social Interaction	-0.013	0.958	-0.256	0.277	-0.105	0.660	0.034	0.888
LORQv3 Section2	-0.278	0.357	-0.364	0.222	0.155	0.613	0.130	0.672
EORTC-ELD-14 JointStiffness	-0.249	0.290	0.090	0.705	-0.384	0.095	0.020	0.935
EORTC-ELD-14 Mobility	-0.388	0.091	-0.359	0.120	-0.438	0.054	-0.008	0.974
EORTC-ELD-14 FamilySupport	-0.247	0.295	-0.094	0.692	-0.559	0.010	0.238	0.312
EORTC-ELD-14 Worries about others	-0.546	**0.013**	-0.389	0.090	-0.363	0.115	-0.174	0.464
EORTC-ELD-14 Future worries	-0.365	0.114	-0.212	0.369	-0.316	0.174	-0.167	0.482
EORTC-ELD-14 Maintaining Purpose	0.067	0.779	0.008	0.975	0.211	0.372	-0.271	0.247
EORTC-ELD-14 Burden of illness	-0.175	0.460	0.028	0.907	-0.321	0.167	-0.002	0.993

EORTC-C30 Physical Functioning	0.672	0.001	0.551	**0.012**	0.374	0.104	0.098	0.681
EORTC-C30 Role Functioning	0.262	0.264	0.226	0.338	0.395	0.085	-0.031	0.897
EORTC-C30 Emotional Functioning	0.074	0.757	0.136	0.569	0.432	0.057	0.116	0.626
EORTC-C30 Cognitive Functioning	0.194	0.413	0.161	0.497	0.611	**0.004**	0.004	0.986
EORTC-C30 Social Functioning	0.297	0.203	0.251	0.287	0.343	0.138	0.551	**0.012**
EORTC-C30 Dyspnoe	-0.025	0.917	0.242	0.304	-0.034	0.887	-0.055	0.816
EORTC-C30 Insomnia	0.111	0.643	0.229	0.333	0.011	0.964	0.091	0.703
EORTC-C30 Appetite loss	-0.116	0.627	-0.408	0.074	-0.551	0.012	-0.167	0.481
EORTC-C30 Nausea	-0.548	**0.012**	-0.668	**0.001**	-0.332	0.152	-0.030	0.900
EORTC-C30 Constipation	0.024	0.919	-0.154	0.516	-0.360	0.119	-0.234	0.320
EORTC-C30 Diarrhoe	0.027	0.911	-0.311	0.181	0.208	0.380	-0.085	0.721
EORTC-C30 Fatigue	-0.104	0.661	0.056	0.813	0.069	0.771	-0.327	0.159
EORTC-C30 Pain	-0.174	0.462	-0.037	0.876	-0.084	0.724	-0.267	0.256
EORTC-C30 Financial difficulties	-0.137	0.566	-0.273	0.245	-0.432	0.057	-0.346	0.135
EORTC-C30 QoL/Global health	0.419	0.066	0.432	0.057	0.146	0.538	0.350	0.130
