# Supplementary material for: Comprehensive Geriatric Assessment and Quality of Life Aspects in Patients with Recurrent/Metastatic Head and Neck Squamous Cell Carcinoma (HNSCC)
Source: J Clin Med. 2023 Sep 3;12(17):5738. doi: 10.3390/jcm12175738 (PMC10488489; doi:10.3390/jcm12175738)
Supplement: Supplementary file 1 [file jcm-12-05738-s001.zip › Table S8.pdf]

Table S8. T2 Spearman rank correlations (r_s) for associations between CGA parameters and questionnaire subscales. Significant p-values ($\alpha \leq .05$) are marked bold.

CGA Parameter	CCI		G8		ECOG		TUG	
Questionnaire	r_s	p	r_s	p	r_s	p	r_s	p
HADS-A	0.139	0.594	-0.470	0.057	0.292	0.255	0.012	0.963
HADS-D	0.234	0.365	-0.666	0.003	0.261	0.311	0.242	0.349
LORQv3 total	0.445	0.084	-0.252	0.347	0.035	0.897	-0.041	0.880
LORQv3 section1	0.341	0.196	-0.465	0.070	0.367	0.162	0.096	0.725
LORQv3 Oral Function	0.392	0.133	-0.317	0.231	0.412	0.113	0.014	0.960
LORQv3 Orofacial Appearance	0.051	0.850	-0.427	0.099	-0.057	0.834	0.237	0.378
LORQv3 Social Interaction	0.404	0.121	-0.420	0.105	0.114	0.110	0.178	0.509
LORQv3 Section2	-0.204	0.661	0.327	0.474	-0.722	0.067	-0.577	0.175
EORTC-ELD-14 JointStiffness	0.411	0.101	-0.087	0.740	0.299	0.244	-0.063	0.810
EORTC-ELD-14 Mobility	-0.286	0.284	0.435	0.092	-0.626	**0.010**	-0.511	**0.043**
EORTC-ELD-14 FamilySupport	0.580	**0.015**	-0.585	**0.014**	0.601	**0.011**	0.310	0.226
EORTC-ELD-14 Worries about others	-0.228	0.379	0.368	0.147	-0.280	0.277	-0.135	0.605
EORTC-ELD-14 Future worries	0.300	0.242	-0.547	**0.023**	0.396	0.116	0.024	0.926
EORTC-ELD-14 Maintaining Purpose	0.308	0.230	-0.222	0.393	0.070	0.789	0.024	0.926
EORTC-ELD-14 Burden of illness	-0.259	0.316	-0.193	0.458	0.352	0.166	0.425	0.089
EORTC-C30 Physical Functioning	0.278	0.281	-0.488	**0.047**	0.586	**0.013**	0.335	0.188
EORTC-C30 Role Functioning	0.260	0.331	-0.249	0.353	0.138	0.610	0.247	0.356
EORTC-C30 Emotional Functioning	0.250	0.350	-0.330	0.211	0.497	**0.050**	0.222	0.408
EORTC-C30 Cognitive Functioning	0.524	**0.037**	-0.677	**0.004**	0.557	**0.025**	0.462	0.072
EORTC-C30 Social Functioning	0.188	0.486	0.034	0.902	0.389	0.137	-0.063	0.816
EORTC-C30 Dyspnoe	0.000	1.000	-0.196	0.467	0.187	0.488	-0.205	0.446

EORTC-C30 Insomnia	0.330	0.212	-0.571	**0.021**	0.512	**0.043**	0.073	0.789
EORTC-C30 Appetite loss	0.105	0.697	-0.320	0.227	0.175	0.518	0.558	**0.025**
EORTC-C30 Nausea	0.364	0.165	-0.379	0.147	0.426	0.100	0.217	0.420
EORTC-C30 Constipation	0.209	0.437	0.154	0.568	0.060	0.824	-0.372	0.155
EORTC-C30 Diarrhoe	-0.532	**0.034**	0.682	**0.004**	-0.638	**0.008**	-0.335	0.205
EORTC-C30 Fatigue	-0.354	0.179	0.388	0.138	-0.312	0.239	-0.351	0.183
EORTC-C30 Pain	-0.516	**0.041**	0.372	0.156	-0.436	0.091	-0.041	0.879
EORTC-C30 Financial difficulties	-0.357	0.174	0.592	**0.016**	-0.363	0.167	-0.408	0.116
EORTC-C30 QoL/Global health	-0.416	0.109	0.230	0.392	-0.471	0.066	-0.220	0.412

**CGA Parameter**	**ADL**		**IADL**		**MMSE**		**MNA**	
**Questionnaire**	r_s	*p*	r_s	*p*	r_s	*p*	r_s	*p*
HADS-A	0.156	0.550	-0.129	0.622	-0.149	0.567	-0.265	0.305
HADS-D	-0.304	0.235	-0.395	0.117	-0.264	0.306	-0.533	**0.028**
LORQv3 total	0.229	0.393	-0.048	0.860	0.264	0.324	-0.189	0.482
LORQv3 section1	0.091	0.737	-0.271	0.309	0.120	0.658	-0.574	**0.020**
LORQv3 Oral Function	0.088	0.745	-0.233	0.386	0.216	0.421	-0.448	0.082
LORQv3 Orofacial Appearance	0.058	0.831	-0.141	0.602	-0.021	0.940	-0.444	0.085
LORQv3 Social Interaction	-0.077	0.778	-0.470	0.066	-0.042	0.879	-0.540	**0.031**
LORQv3 Section2	0.636	0.124	0.879	**0.009**	0.073	0.877	0.541	0.210
EORTC-ELD-14 JointStiffness	-0.282	0.272	-0.228	0.379	-0.169	0.518	-0.089	0.734
EORTC-ELD-14 Mobility	0.574	**0.020**	0.568	**0.022**	0.705	**0.002**	0.366	0.163
EORTC-ELD-14 FamilySupport	-0.388	0.124	-0.591	**0.012**	-0.397	0.114	-0.650	**0.005**
EORTC-ELD-14 Worries about others	0.280	0.277	0.355	0.162	0.308	0.229	0.174	0.505
EORTC-ELD-14 Future worries	-0.054	0.835	-0.285	0.267	-0.141	0.590	-0.623	**0.008**
EORTC-ELD-14 Maintaining Purpose	-0.017	0.947	-0.151	0.564	-0.023	0.929	-0.243	0.347
EORTC-ELD-14 Burden of illness	-0.089	0.733	-0.273	0.289	-0.183	0.482	-0.097	0.711

EORTC-C30 Physical Functioning	-0.508	**0.037**	-0.655	**0.004**	-0.299	0.244	-0.517	**0.033**
EORTC-C30 Role Functioning	-0.245	0.359	-0.435	0.092	-0.294	0.270	-0.287	0.282
EORTC-C30 Emotional Functioning	-0.340	0.197	-0.343	0.194	-0.477	0.062	-0.187	0.489
EORTC-C30 Cognitive Functioning	-0.412	0.113	-0.659	**0.005**	-0.410	0.115	-0.712	**0.002**
EORTC-C30 Social Functioning	-0.185	0.493	-0.175	0.516	-0.089	0.743	-0.024	0.930
EORTC-C30 Dyspnoe	0.194	0.473	0.009	0.974	-0.096	0.724	0.075	0.782
EORTC-C30 Insomnia	-0.097	0.721	-0.476	0.062	-0.191	0.478	-0.472	0.065
EORTC-C30 Appetite loss	-0.302	0.256	-0.504	**0.047**	-0.444	0.085	-0.205	0.447
EORTC-C30 Nausea	-0.185	0.492	-0.355	0.177	-0.260	0.330	-0.270	0.311
EORTC-C30 Constipation	0.315	0.235	0.187	0.489	0.378	0.149	0.215	0.425
EORTC-C30 Diarrhoe	0.409	0.115	0.543	**0.030**	0.469	0.067	0.776	**0.000**
EORTC-C30 Fatigue	0.401	0.124	0.451	0.079	0.311	0.241	0.263	0.325
EORTC-C30 Pain	0.099	0.716	0.372	0.156	0.090	0.740	0.413	0.111
EORTC-C30 Financial difficulties	0.272	0.308	0.438	0.090	0.428	0.098	0.706	**0.002**
EORTC-C30 QoL/Global health	0.223	0.406	0.344	0.192	0.195	0.470	0.474	0.064
